# Detection of Lung Cancer via Blood Plasma and ^1^H-NMR Metabolomics: Validation by a Semi-Targeted and Quantitative Approach Using a Protein-Binding Competitor

**DOI:** 10.3390/metabo11080537

**Published:** 2021-08-12

**Authors:** Elien Derveaux, Michiel Thomeer, Liesbet Mesotten, Gunter Reekmans, Peter Adriaensens

**Affiliations:** 1 Faculty of Medicine and Life Sciences, Hasselt University, Martelarenlaan 42, B-3500 Hasselt, Belgium; elien.derveaux@uhasselt.be (E.D.); michiel.thomeer@uhasselt.be (M.T.); liesbet.mesotten@zol.be (L.M.); 2 Department of Respiratory Medicine, Ziekenhuis Oost-Limburg, Schiepse Bos 6, B-3600 Genk, Belgium; 3 Department of Nuclear Medicine, Ziekenhuis Oost-Limburg, Schiepse Bos 6, B-3600 Genk, Belgium; 4 Applied and Analytical Chemistry, Institute for Materials Research, Hasselt University, Agoralaan 1—Building D, B-3590 Diepenbeek, Belgium; gunter.reekmans@uhasselt.be

**Keywords:** lung cancer diagnosis, NMR metabolomics, metabolite profile, maleic acid internal standard, protein-binding competitor, OPLS-DA classification

## Abstract

Metabolite profiling of blood plasma, by proton nuclear magnetic resonance (^1^H-NMR) spectroscopy, offers great potential for early cancer diagnosis and unraveling disruptions in cancer metabolism. Despite the essential attempts to standardize pre-analytical and external conditions, such as pH or temperature, the donor-intrinsic plasma protein concentration is highly overlooked. However, this is of utmost importance, since several metabolites bind to these proteins, resulting in an underestimation of signal intensities. This paper describes a novel ^1^H-NMR approach to avoid metabolite binding by adding 4 mM trimethylsilyl-2,2,3,3-tetradeuteropropionic acid (TSP) as a strong binding competitor. In addition, it is demonstrated, for the first time, that maleic acid is a reliable internal standard to quantify the human plasma metabolites without the need for protein precipitation. Metabolite spiking is further used to identify the peaks of 62 plasma metabolites and to divide the ^1^H-NMR spectrum into 237 well-defined integration regions, representing these 62 metabolites. A supervised multivariate classification model, trained using the intensities of these integration regions (areas under the peaks), was able to differentiate between lung cancer patients and healthy controls in a large patient cohort (*n* = 160), with a specificity, sensitivity, and area under the curve of 93%, 85%, and 0.95, respectively. The robustness of the classification model is shown by validation in an independent patient cohort (*n* = 72).

## 1. Introduction

Metabolomics, or the study of low-molecular-weight molecules in biofluids, such as blood plasma, serum, and urine, offers great potential to answer critical clinical research questions [1,2,3,4,5,6,7,8,9]. A frequently used technique to analyze these complex biological samples is nuclear magnetic resonance (NMR) spectroscopy [10,11,12,13]. This analytical tool allows quantitative data collection in a robust and highly reproducible way, when a standardized procedure is used [14,15,16,17,18]. Although NMR metabolomics is still an upcoming field, several research groups have developed analytical protocols based on different sample preparation conditions, to establish a metabolite profile or fingerprint [10,18,19,20,21]. Next to mass-spectrometry, multiple metabolite profiling studies are accomplished by means of proton (^1^H)-NMR metabolomics, since it allows the quantification of all the metabolites in a single run.

Blood plasma inherently contains macromolecules, such as proteins and lipoproteins, of which the signals may overlap with some metabolite signals in the ^1^H-NMR spectrum and, by this, mask the presence or the correct concentration of those metabolites [22,23,24]. To overcome this issue, some research groups proposed protocols to remove the (lipo)proteins, such as ultrafiltration [25,26,27] or precipitation with organic solvents [26,27,28]. However, such sample treatments are rather laborious and time consuming, and, most importantly, are generally prone to low reproducibility. An alternative approach to get rid of these macromolecular-related signals in the ^1^H-NMR spectrum can be found in the use of the Carr–Purcell–Meiboom–Gill (CPMG) pulse sequence, which allows attenuation of the broad signals of macromolecules that have short T_2_ relaxation decay times, with minimal influence on the signals of the small metabolites that have much longer T_2_ decay relaxation times [10,18,29]. Numerous clinical NMR metabolomics studies are reported that use the CPMG pulse sequence [1,3,4,5,19,30,31,32,33].

In ^1^H-NMR metabolomics, the addition of deuterated trimethylsilyl-2,2,3,3-tetradeuteropropionic acid (TSP) is frequently used to calibrate the chemical shift ppm scale, because the trimethylsilyl group gives rise to a strong, sharp and selective upfield singlet signal. For blood plasma, however, the binding of TSP to human serum albumin (HSA) causes fluctuations in the chemical shift of the TSP signal [34]. Moreover, the TSP signal cannot be used as an internal standard for quantification either, because of its high binding affinity to HSA, as is reported here and by several research groups [34,35,36]. Nevertheless, the addition of 4 mM TSP to blood plasma samples has a tremendous advantage that is not yet described in the literature, and is explained in detail in this paper.

Dividing the ^1^H-NMR spectrum of human blood plasma into integration regions offers the possibility of using these regions’ integration values as variables in a multivariate statistical model with clinical applicability. The procedure to define these integration regions is, however, often variable. Binning is a fast and easily automated method, in which the spectrum is segmented into equally sized regions or so-called bins. A disadvantage of this technique is the loss of statistical power, due to the fragmentation of peaks. Therefore, other research groups invested in the development of adaptive binning methods [37,38]. Several advances have been made in automated peak picking, for example, by applying algorithms for metabolite peak detection, based on the chemical shift and J-coupling pattern, by which variable-sized integration regions can be defined [39,40,41]. These methods allow rapid and automated data collection from raw ^1^H-NMR spectra for statistical analysis. However, J-coupling patterns or reporter signals are often not detected when the metabolite concentration is low, leaving the spectra with unrecognized residual signals and even incomplete datasets. Another approach, especially when interest lies in disease diagnosis as well as in studying the changing metabolism in the search for therapy development, is a targeted metabolite spiking approach. This enables the identification of metabolites that are most influenced by the disease, and therefore contribute most to the differentiating power of the statistical model [42].

In this paper, metabolite spiking is applied to accurately determine well-understood integration regions in the plasma ^1^H-NMR spectrum. In addition, the influence of adding 4 mM TSP on the chemical shift and signal intensity of the plasma metabolites is evaluated, in order to accurately measure these integration regions. Additionally, the affinity of HSA for TSP and maleic acid (MA) is described, leading to the introduction of MA as an internal standard for plasma metabolite quantification, without the need for protein precipitation.

While the dataset of some studies is rather limited, the robustness of our protocol is demonstrated and validated in a large patient cohort of lung cancer patients and healthy controls.

## 2. Results

The following paragraphs demonstrate that maleic acid (MA) can be used as a reliable internal standard in NMR metabolomics of human plasma, when combined with the addition of 4 mM trimethylsilyl-2,2,3,3-tetradeuteropropionic acid (TSP). Firstly, it will be demonstrated that certain plasma metabolites, as well as MA, bind to human serum albumin (HSA) if no TSP is added. As a result, a sample-to-sample variation in HSA concentration leads to errors in the integration values (area under the peaks) of the signals of these metabolites, since the HSA-bound fractions are underestimated, due to their short T_2_ relaxation decay time. Secondly, it will be shown that TSP has a much higher affinity for HSA, and therefore can be used as an HSA-binding competitor, avoiding unwanted metabolite binding.

### 2.1. TSP Has a High Affinity for HSA and Ensures Dissociation of Protein-Bound Metabolites

**Figure 1:**
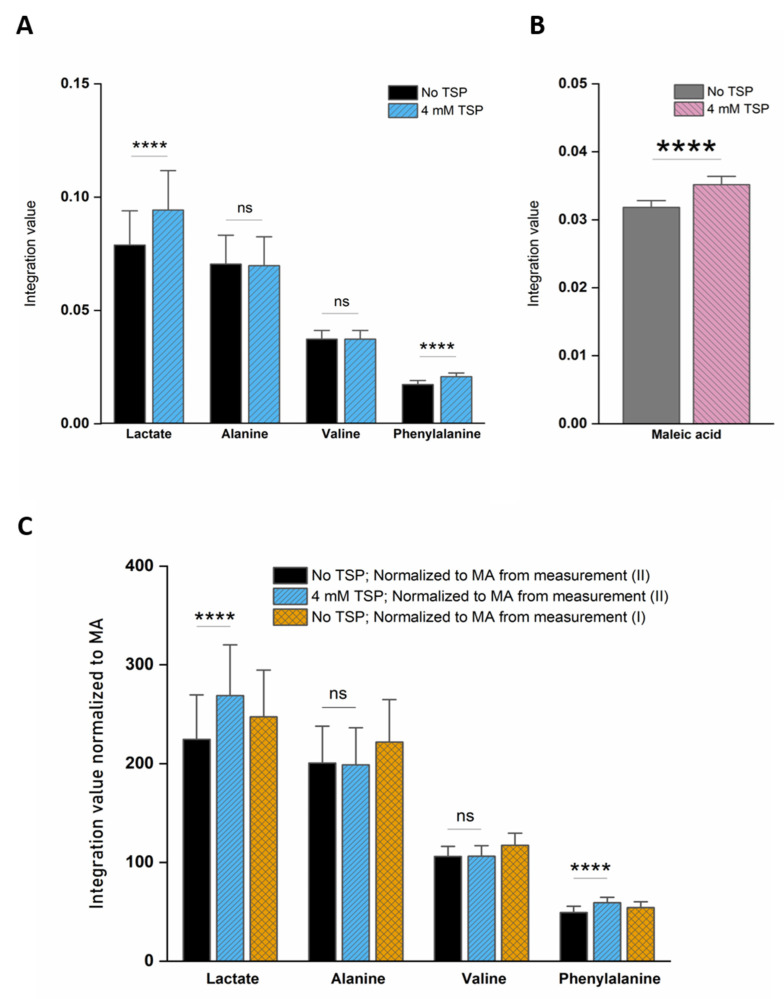
Non-IS-normalized integration values with standard deviation (from 12 different plasma samples) for (**A**) lactate, phenylalanine, alanine, and valine and (**B**) maleic acid in human plasma samples without (I; black bars) and with 4 mM TSP (II; blue bars). Measurement (I) was immediately followed by measurement (II) under fully identical measuring conditions. Whereas the integration value for alanine and valine signals remains unchanged after TSP addition, the integration values increase significantly for lactate, phenylalanine, and maleic acid. (**C**) Integration values of measurement (I) can be overestimated or underestimated when normalized towards MA without addition of TSP (yellow bars) because several metabolites as well as MA itself bind to HSA. Correct MA-normalized integration values for all metabolites are only obtained when 4 mM TSP is present (blue bars). MA: maleic acid; ns: not significant; TSP: trimethylsilyl-2,2,3,3-tetradeuteropropionic acid; (**** *p* < 0.0001).

Since HSA-bound TSP has a short T_2_ relaxation decay time compared to free TSP, the detected TSP signal intensity (area under the peak) will be underestimated and will depend on the amount of HSA that is present in the plasma sample (the bound TSP fraction will not, or only partially, be detected). Therefore, the TSP signal intensity no longer reflects the known amount of TSP that is added during the sample preparation. Analogous to this, some metabolites also bind to HSA, resulting in signal intensities that do not reflect their total concentration, but rather the HSA-unbound fraction. However, since TSP shows a much higher affinity for HSA than these metabolites, the addition of TSP to the plasma sample leads to dissociation of these metabolites from HSA. Identification of the metabolites that are influenced by TSP–HSA binding was performed by evaluating changes in the chemical shift and signal intensity in the ^1^H-NMR spectrum, upon adding increasing amounts of TSP to a series of identical plasma reference samples (identical samples taken from a plasma pool). Increasing the amount of TSP shows that stable signal intensities and chemical shifts are reached for all the signals in the spectrum, after the addition of 4 mM TSP. Higher TSP concentrations have no further influence on the integration values and peak positions (Figure S1). These findings follow the suggestions that were reported by Barrilero et al., who used 3 mM TSP to release HSA-binding metabolites [43]. In a second experiment, the same observation was made for 12 different plasma samples, obtained from 12 different donors, upon the addition of 4 mM TSP. Hereto, 12 samples were prepared and measured without (I) and with (II) the addition of 4 mM TSP (10 µL of TSP-containing buffer solution was added to the NMR tube after the first measurement). Note that measurement (I) was immediately followed by measurement (II), under completely identical NMR measuring conditions, allowing a direct comparison of the peak intensities, which are still not normalized towards an internal standard (non-IS-normalized). Figure 1A illustrates this nicely, for two affected metabolites, lactate and phenylalanine, versus two non-affected metabolites, alanine and valine. The observed standard deviations (SD) are due to the intrinsic sample-to-sample metabolite variations between the 12 donors. The metabolites that fully dissociate from HSA, upon the addition of 4 mM TSP, are identified as acetate, acetoacetate, lactate, oxaloacetate, phenylalanine, pyruvate, 3-methyl-2-oxobutyrate, and β-hydroxybutyrate, next to a non-identified metabolite. Other metabolites, such as alanine and valine, do not bind to HSA, and are therefore not influenced by the addition of TSP. These experiments, on a series of plasma samples from different donors, confirm the reproducibility of the effect of adding 4 mM TSP. Since alanine does not bind to HSA, and its doublet signal representing its methyl group is a sharp and non-overlapping signal, it can efficiently serve as an internal chemical shift reference. In this study, the upfield signal of the alanine doublet is fixed at 1.4938 ppm. This reference ppm value is the average position of the upfield alanine peak in the 12 spectra of the different plasma samples, after setting the TSP peak at 0.00 ppm.

### 2.2. Maleic Acid as an Internal Standard for Quantification in NMR Metabolomics of Plasma Containing 4 mM TSP

Undoubtedly, TSP is no fitting candidate to serve as a reference for quantification, because of its high binding affinity to HSA. To enable quantification of the plasma metabolites, a known concentration of a useful internal standard should be added to the buffer stock solution. The essential criteria for internal standard selection include that its signal is sharp and not overlapping with other signals, and thus easy to integrate. In the proposed methodology, MA is used, since it is well known in analytical chemistry. MA can be purchased with excellent purity, easily dried, and has a distinct solubility in D_2_O, making it an attractive internal standard for quantitative NMR [44]. The addition of MA to plasma samples, in buffer pH 7.4, gives rise to a sharp singlet around 6 ppm, which is a region where no other metabolite signals appear.

**Figure 2:**
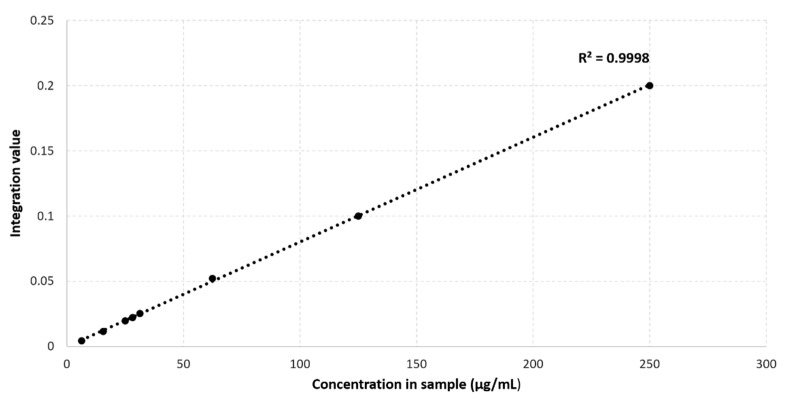
Calibration curve of maleic acid in human plasma containing 4 mM TSP.

Figure 1B shows that the addition of 4 mM TSP to the 12 different plasma samples also results in an increase in the MA intensity. This indicates that MA also binds to HSA, however, with a much lower affinity for HSA than the competing TSP. A small relative standard deviation (%RSD) value of only 3.54% is found, originating from the sample preparation, NMR measurement, and integration. Figure 2 shows an almost perfect linear calibration curve that was obtained by means of eight identical reference plasma pool samples, containing 4 mM TSP and a different, but known, amount of MA. The linear behavior with an R^2^ value of 0.9998, demonstrates the absence of association between MA and HSA under these conditions.

**Figure 3:**
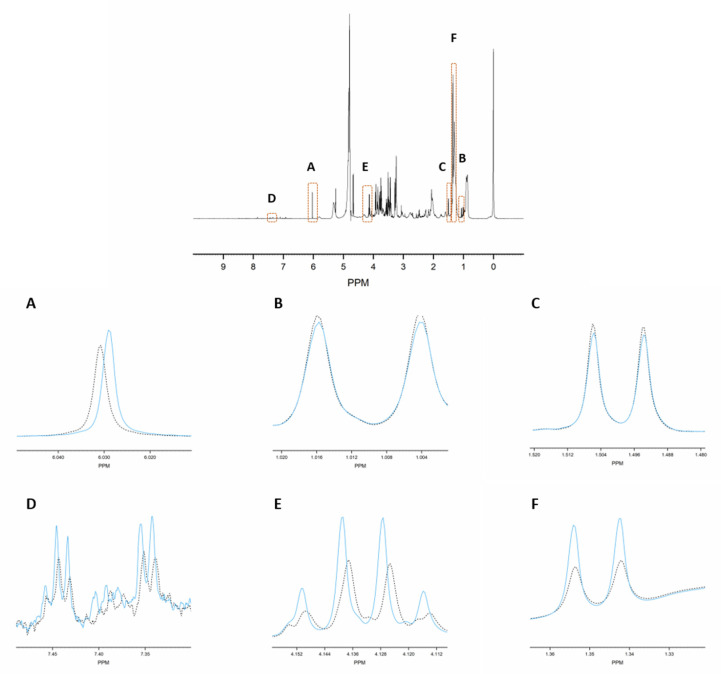
The effect of adding TSP on the chemical shift and intensity of some metabolite signals in human plasma. The signals before addition of TSP (dotted black lines) and after addition of 4 mM TSP (solid blue lines). (**A**): maleic acid; (**B**): valine; (**C**): alanine; (**D**): phenylalanine; (**E**,**F**): lactate.

Consequently, MA is an ideal internal standard when used in combination with 4 mM TSP. Figure 1C demonstrates that without TSP, the MA-normalized integration values are being overestimated or underestimated (yellow bars), because several metabolites, as well as MA itself, bind to HSA. Only in the presence of 4 mM TSP are correct MA-normalized integration values (and so absolute metabolite concentrations) obtained (blue bars). Figure 3 provides a visual overview of the influence of adding 4 mM TSP on the metabolite signals, as discussed above. Altogether, these results indicate that MA is an excellent internal standard for quantification in plasma NMR metabolomics, if combined with the addition of 4 mM of the strong HSA-binding competitor TSP.

### 2.3. Spiking with 62 Known Metabolites Results in 237 Well-Defined Integration Regions

Large metabolomic databases that provide information about human metabolites and their NMR characteristics, are powerful, accessible tools that can guide new metabolomics research [45,46]. However, it is well known that ‘external’ conditions, such as pH, ion strength, sample concentration, and temperature, influence the ^1^H-NMR chemical shifts of human blood plasma metabolites [47,48,49]. Additionally, ‘intrinsic’ conditions, such as the donor-specific HSA concentration, will not only lead to chemical shift changes in the signals of the HSA-bound metabolites, but also to a sample-to-sample dependent underestimation of the amount of such HSA-bound metabolites. In our proposed measuring protocol, the choice of the doublet signal of alanine, serving as chemical shift reference at 1.4938 ppm, was prompted by the fact that it is not influenced by the HSA concentration. A previous study already showed that metabolite identification, using a spiking approach, provides a robust way to divide the ^1^H-NMR spectrum into well-defined integration regions, for the collection of a spectral dataset that allows metabolic profiling of human plasma [42]. Efforts are hereby made to define the integration regions that represent single metabolites, as much as possible. Therefore, spiking experiments were performed for 62 metabolites, using the same sample preparation, NMR measurement conditions, and processing as described above and in the experimental section. Based on this spiking information, the plasma ^1^H-NMR spectrum was divided into 237 well-defined integrations regions, each representing one or more metabolites. All 62 metabolites, with their chemical shifts, multiplicity, and J-coupling values, are summarized in Table 1, together with their assigned integration region numbers (VAR 001–VAR 237). Table S1 shows the metabolite composition of each integration region, and its start and end ppm values.

### 2.4. The Proposed Methodology Shows a High Robustness Level

To evaluate the robustness of the proposed method, plasma from multiple donors was combined, to obtain a large reference plasma pool from which identical plasma aliquots of 400 µL were taken and stored at −80 °C. Twelve identical NMR samples were prepared from 12 of these aliquots, measured, and processed using the above conditions. The integration values of all 237 integration regions were normalized to that of the MA signal. The MA signal showed a %RSD of only 1.98%. Table S2 demonstrates that the intrasample variability is <10% for almost all the regions, with a large majority of the regions showing even a %RSD <5%. Only nine regions showed a higher variability, they are as follows: VAR 001 (formate, singlet), VAR 009 (3-methylhistidine, singlet), VAR 021 (fumarate, singlet), VAR 022 (uridine, doublet), VAR 024 (allantoin, singlet), VAR 027 (mannose, doublet), VAR 029 (carnitine, multiplet), VAR 030 (non-identified metabolite), and VAR 031 (non-identified metabolite). The higher intra-variability for these regions can be explained by (i) the influence of the neighboring water peak (VAR 027, VAR 029, VAR 030, and VAR 031) and (ii) the very low signal intensity, resulting in a poor signal/noise ratio (VAR 001, VAR 009, VAR 021, VAR 022, and VAR 024).

### 2.5. Method Validation: The Proposed Method Allows Differentiation between Lung Cancer Patients and Healthy Controls in a Large Study Cohort

**Figure 4:**
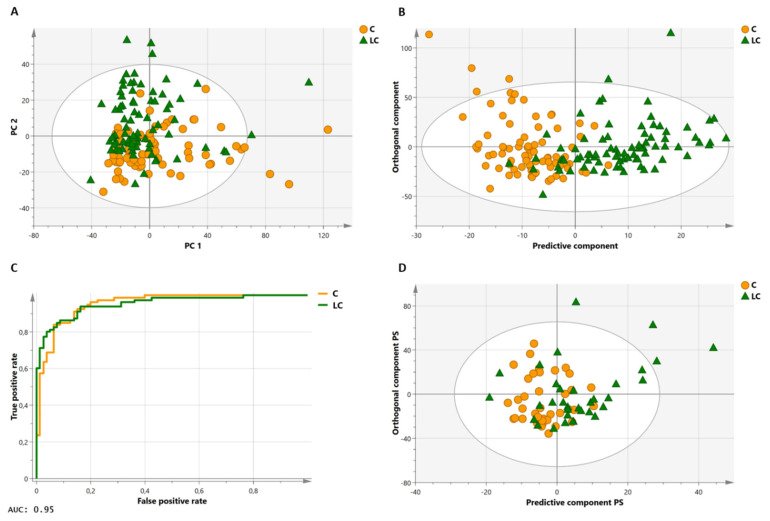
Multivariate statistical analyses on a large lung cancer cohort to validate the proposed methodology. (**A**) Principal component analysis (PCA) of the training cohort showing clustering of the two groups. Using only the first two principal components, the PCA model shows an R^2^ value of 0.775 and a Q^2^ value of 0.746. (**B**) Orthogonal partial least squares discriminant analysis (OPLS-DA) classifier of the training cohort consisting of 80 controls and 80 lung cancer patients. The training model discriminates between the two groups with 93% specificity and 85% sensitivity. (**C**) The receiver operating characteristic (ROC) curve of the trained classifier shows high predictive accuracy with an AUC of 0.95. (**D**) Validation of the OPLS-DA classifier on an independent validation cohort consisting of 38 controls and 34 lung cancer patients. The validation cohort discriminates between the two groups with a specificity and sensitivity of 74%. AUC: area under the curve; C: controls; LC: lung cancer patients; PC: principal component; PS: predicted scores.

To validate the proposed methodology and demonstrate its discriminative potential, plasma samples of 141 lung cancer patients and 135 healthy controls were measured and analyzed, as described above. Hemolytic plasma samples (*n* = 44) were excluded from the analysis. Although our group previously demonstrated that both severe (free hemoglobin concentration ≥ 100 mg/dL) and mild (free hemoglobin concentration ≥ 10 mg/dL) hemolysis did not influence the plasma metabolite profile [50], it was decided to (not yet) include these 44 hemolytic samples in this proof-of-principle study, to ensure correct validation of the proposed methodology (the sample cohorts are still quite large without the hemolytic samples). Table 2 shows the clinical characteristics of the lung cancer patients and healthy controls that were used in the training and validation cohort datasets. The intensities (areas under the peaks) of the well-defined integration regions (Table S1) serve as variables in the multivariate statistical models. These intensities were normalized to the integration value of the MA standard, of which the chemical shift and linewidth was closely monitored for all the measurements (no abnormalities were observed). The first statistical orthogonal partial least squares discriminant analysis (OPLS-DA) training model was constructed, by using samples of 80 lung cancer patients and 80 healthy controls, excluding the 9 variables showing a high intrasample variability (%RSD >10%—see also Table S2), and 7 variables showing a high intersample variability (%RSD >30%: VAR 080, VAR 209, VAR 215, VAR 216, VAR 217, VAR 218, and VAR 237), in both the cancer and control group (Figure S2). Starting from this model (constructed with 221 variables), further data reduction was performed, by excluding the variables that, based on their loading values and their standard errors that were calculated by cross-validation (jack-knifing), showed little or no significant contribution to the model (143 variables). The unsupervised PCA plot, obtained using this reduced dataset of 78 variables (221 minus 143 variables), shows a clear distinctive trend between the two groups (Figure 4A). The same 78 remaining variables were used for training a supervised OPLS-DA classifier, showing R^2^X(cum) and R^2^Y(cum) model parameters of, respectively, 0.861 and 0.581, and a Q^2^(cum) value of 0.364 (Figure 4B). This model allows discrimination between the lung cancer patients and healthy controls, with a specificity of 93% and a sensitivity of 85%. Moreover, the receiver operating characteristic (ROC) curve shows an area under the curve (AUC) of 0.95 (Figure 4C). Strengthened by the PCA analysis, this all demonstrates that the group separation in the supervised classification model is not based on over-fitting due to ‘noisy’ data. Further support is found in a PCA analysis, obtained by using the reduced dataset (78 variables) and all 232 subjects (of the training and validation cohort together), also showing the distinctive trend between the lung cancer patients and controls (Figure S3). Moreover, permutation testing of the trained classifier resulted in R^2^ and Q^2^ values of, respectively, 0.206 and −0.339, again supporting the strength of the model (Figure S4). Furthermore, testing the trained model on an independent validation cohort of 34 lung cancer patients and 38 controls confirms its validity. Group separation remains apparent in the validation model, with a specificity and sensitivity of 74% for both (Figure 4D).

### 2.6. Identification of Metabolites Contributing Strongest to the Model Reveals Reprogrammed Biochemical Pathways in Lung Cancer

Table S3 summarizes the 30 variables with a VIP (variable importance in projection) value >0.80, and indicates whether a decreased or increased integration value (metabolite concentration) is observed for the lung cancer patients compared to the healthy controls. The integration values of 13 variables show a decrease in lung cancer patients, and could be attributed to the lipid signals of fatty acid chains (FAC), phosphatidylcholines (PC), or sphingomyelins (SM). The integration values of the other 17 most discriminative variables are increased in the lung cancer patients. After careful interpretation of the NMR spectra, as described in the Section 4, the metabolites that are responsible for this increase are identified as glucose, isoleucine, leucine, glycerol, and isopropanol.

## 3. Discussion

This study presents the successful development of a robust quantitative ^1^H-NMR metabolomics method and its validation, by showing differentiation between large cohorts of lung cancer patients and healthy controls, based on their plasma metabolite profile.

In order to quantify plasma metabolites, some studies describe the use of internal standards, such as formic acid [10,51,52] or 4,4-dimethyl-4-silapentane-1-ammonium trifluoroacetate (DSA) [53]. However, when future studies demand for an absolute determination of metabolite concentrations, the standard should be very pure and water-free (to avoid a contribution of bounded water and impurities to the analytically weighed amount of the standard), which will be difficult for these standards. In contrast, maleic acid (MA) is a commonly used analytical standard, which is easily dried in a vacuum oven, and can be purchased with excellent purity, making it an ideal and reliable internal standard for plasma metabolite quantification.

This study demonstrates, for the first time, that combining MA and 4 mM TSP, as a strong human serum albumin (HSA) binding competitor, allows an accurate determination of the plasma metabolite concentrations, because the binding of metabolites and MA to HSA is prevented. Under these conditions, the upfield peak of the methyl doublet of the non-HSA-binding metabolite alanine, having a large J-coupling value of 7.2 Hz, is an ideal candidate to calibrate the chemical shift ppm scale, since the doublet lines are baseline resolved. Moreover, plasma HSA comprises 50% to 60% of the total plasma proteins [54], and varies between individuals, hereby explaining the sample-to-sample differences in the chemical shift and intensity of several plasma metabolites in the ^1^H-NMR spectrum, when TSP is added in only a small amount or not at all. The use of MA as internal standard was recently also reported by Bliziotis et al. [55], but the effect of TSP on the HSA–MA binding was not taken into account. Gowda et al. recently also verified the use of MA (or fumaric acid) for blood plasma samples, and proposed to use it as an internal standard in combination with protein precipitation [56].

Other reported methods that (partly) release HSA-bounded metabolites by using an HSA competitor, include the addition of, e.g., fatty acids [24] or SDS [57]. However, the addition of these compounds will give rise to additional overlapping signals in the spectrum, which is not the case for the upfield TSP signal around 0.00 ppm. Furthermore, in contrast to this protocol that allows the inclusion of the contribution of different lipid types to the metabolite profile, as shown in Table S1, the addition of fatty acids will hinder this possibility.

Where the ^1^H-NMR metabolomics spectra often encounter signal assignment uncertainties, due to overlapping metabolite signals, the proposed methodology resolves this issue by performing selective metabolite spiking experiments (instead of using only library-based identification). All the signals of 62 metabolites are hereby identified, and consequently, 237 well-understood integration regions are defined that can serve as accurate variables to establish a reliable metabolite profile, or fingerprint, of an individual that allows the construction of multivariate statistical models for, e.g., disease diagnosis and therapy follow-up, as well as deeper investigation of the metabolic pathways related to the disease. By crucial validation of the proposed methodology in a large patient cohort (*n* = 232), it is shown that the method is robust and enables a clear differentiation between lung cancer patients and healthy controls.

In this study, the metabolite profile is further demonstrated to be of high value for a targeted profiling, enabling immediate backtracking of the critical key metabolites that contribute to a disturbed metabolism in lung cancer diseases. The unraveling of cancer metabolism, with its aberrant biochemical pathways, is classically focused on central carbon metabolism and the support of tumor growth via rapid energy production. Although it was not yet the primary goal of this methodology study, this paper already discusses the role of the metabolites that differentiate the strongest between lung cancer patients and healthy controls in the reprogrammed biochemical pathways. A detailed metabolic pathway interpretation will be the content of a follow-up paper.

Proliferating cancer cells are known to be highly glycolytic in order to meet their metabolic requirements. The elevated plasma glucose levels that are found in lung cancer patients, suggest a compensatory upregulated gluconeogenesis in other tissues, primarily the liver, using lactate that is derived from muscle activity, enabling rapid energy production via glycolysis within the tumor [58]. Interestingly, several studies suggest that the upregulated systemic gluconeogenesis can be supported even more by using lactate originating from fermentation (or aerobic glycolysis) within proliferating cancer cells, hereby creating a cyclic metabolic co-operation between tumor and healthy tissue [59,60,61,62].

The increased plasma glycerol levels can be explained in the same context of fueling the highly proliferating tumor cells. Glycerol, from the bloodstream, can enter the tumor cell and serve as a backbone for fuel biosynthesis (triacylglycerols) and phospholipid membrane formation [63]. In addition, glycerol will be used for gluconeogenesis within cancer cells [63]. Indeed, suggestive results, pointing to an adaptive response of gluconeogenesis within cancer cells upon glucose deprivation, were provided by Leithner et al. [64]. The heterogeneous character of tumors should (always) be taken into account, as more and more evidence indicates a combined appearance of glycolysis and gluconeogenesis, with a flexible difference in flux (enhanced metabolic flexibility) rather than a complete separated character within cells [65,66].

This study also reveals a higher plasma concentration of the branched-chain amino acids (BCAAs) leucine and isoleucine, in lung cancer patients. A previous study highlights the fact that BCAAs can also play an anaplerotic role and fuel the tricarboxylic acid (TCA) cycle via conversion to acetyl-CoA [67]. BCAA-derived acetyl-CoA is also reported to be targeted by histone acetyl transferases (HATs), regulating histone acetylation and hereby stimulating gene expression [68,69].

A reduced level of plasma lipids in lung cancer patients is in accordance with the high need of membrane synthesis by cancer cells. In non-small-cell lung cancer (NSCLC) tumor cells, enzymes such as lipoprotein lipase (LPL) are known to use triacylglycerols (TAGs) and phospholipids (PLs) from the bloodstream to acquire energy for membrane synthesis and tumor proliferation via lipolysis [70,71]. Furthermore, the previously mentioned increased plasma glycerol concentrations support the enhanced lipolysis and TAG catabolism [72].

Isopropanol can be oxidized to acetone by alcohol dehydrogenase (ADH)-type enzymes. However, a disturbed high NADH/NAD^+^ ratio can shift this balance towards reduction, explaining an elevated plasma isopropanol level [73]. While healthy cells can rely on ketone bodies (such as acetone) for energy production via oxidative phosphorylation (OXPHOS), tumor cells show an impaired OXPHOS [74,75]. Thus, a ketotic state might be an unfavorable condition for cancer cells.

Expected future work will involve the inclusion of plasma samples from other collection sites, but if the described sample collection protocol is carefully followed, no problem is expected to combine interlab datasets into one large data matrix. Moreover, if other NMR analysis sites come into play (using spectrometers with the same magnetic field strength), it will be important to first validate the analysis protocol by setting up an interlaboratory ring trial using pooled reference plasma samples, in order to evaluate the repeatability of the results.

## 4. Materials and Methods

### 4.1. Materials

Deuterium oxide (D_2_O, 99.95%, Deutero GMBH, Kastellaun, Germany), di-potassium hydrogen phosphate (K_2_HPO_4_, VWR International LLC, Radnor, PA, USA) and potassium dihydrogen phosphate (KH_2_PO_4_, Sigma-Aldrich, Saint Louis, MO, USA) were used for preparation of the buffer stock solution. Maleic acid (MA, ≥99%, Merck, Darmstadt, Germany) and trimethylsilyl-2,2,3,3-tetradeuteropropionic acid (TSP, 99%, Deutero GMBH) were added to obtain the final measuring buffer. Blood samples were collected in lithium heparin blood collection tubes (BD Vacutainer LH 17 I.U. 6 mL tube), and centrifuged in a swinging bucket centrifuge (Jouan GR 4 22). Plasma samples were centrifuged (fixed rotor Eppendorf centrifuge 5415 R, Hamburg, Germany) and measured using a 600 MHz JEOL NMR spectrometer (JEOL Ltd., Tokyo, Japan). NMR data were processed using JEOL Delta software (version 5.3.1, JEOL Ltd., Tokyo, Japan). SIMCA^®^ software (version 15.0.2, Sartorius AG, Goettingen, Germany) was used to perform the statistical analyses.

### 4.2. Ethics Statement

Samples were collected during the registered trial with study number NCT02024113, which was conducted following the Helsinki Declaration and Good Clinical Practice’s ethical rules. The study protocol was approved by the Medical Ethics Committees of ZOL (Ziekenhuis Oost-Limburg, Campus Sint-Jan, Genk, Belgium) and Hasselt University (Hasselt, Belgium). Signed informed consent was obtained from all participants before inclusion.

### 4.3. Subjects

All blood samples were collected from donors who met the following inclusion criteria: (I) fasted and no medication intake for at least 6 h, (II) fasting blood glucose concentration below 200 mg/dL, and (III) no treatment or history of cancer in the past 5 years. Plasma samples of a plasma pool (multiple donors), further referred to as ‘plasma reference samples’, were used for the maleic acid calibration curve; the metabolite spiking experiments; and the determination of the error on the NMR sample preparation and measurement.

**Figure 5:**
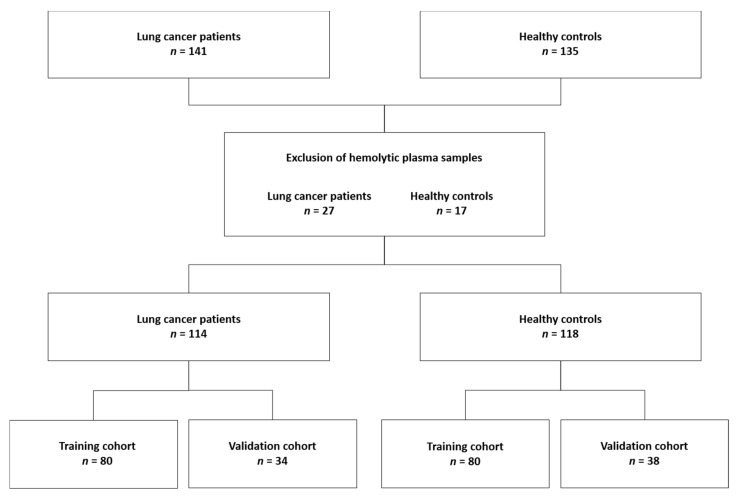
CONSORT diagram of the subjects enrolled in the training (*n* = 160) and validation (*n* = 72) cohort.

*Subjects for the model training and validation.* Plasma samples from 276 donors were analyzed, but hemolytic plasma samples (*n* = 44) were excluded from the dataset. Finally, 160 subjects (80 lung cancer patients and 80 healthy controls) were selected for the training cohort while the remaining 72 subjects (34 lung cancer patients and 38 healthy controls) were used for an independent validation of the trained classification model (Figure 5).

### 4.4. Preanalytical Sample Preparation

*Plasma sample collection and storage.* Plasma samples collected during the NCT02024113 study were retrieved from the University Biobank Limburg (UBiLim) for immediate NMR analysis. All plasma samples were obtained as follows: fasting venous blood was collected and stored at 4 °C within 5 to 10 min. Within 8 h after blood collection, blood samples were centrifuged at 1600× g for 15 min (swinging bucket centrifuge), as described by Louis et al. [2]. Finally, plasma aliquots of 400 µL were transferred into sterile cryovials and stored at −80 °C until NMR analysis.

*Buffer preparation containing TSP and the internal MA standard for quantification.* Stock solutions of 1 M K_2_HPO_4_ and 1 M KH_2_PO_4_ in D_2_O were prepared by dissolving, respectively, 174.18 g/L and 136.09 g/L in D_2_O. The 0.15 M potassium phosphate pH 7.4 buffer used for NMR sample preparation was obtained as follows: 85 mL D_2_O was added to a solution containing 4 mL of the 1 M KH_2_PO_4_ stock and 11 mL of the 1 M K_2_HPO_4_ stock. The final buffer to prepare the plasma samples for NMR measurements was obtained by dissolving 137.82 mg TSP, and 6.25 mg dried maleic acid (MA) as internal standard for quantification in the 0.15 M phosphate buffer pH 7.4, in order to obtain a buffer containing 8 mM TSP and 107.70 µM (62.50 µg/mL) MA.

*Sample preparation*. Before NMR analysis, plasma aliquots were thawed and homogenized using a vortex mixer. After centrifugation at 13,000× g for 4 min at 4 °C (fixed rotor Eppendorf centrifuge), 350 µL plasma was added to 350 µL 0.15 M potassium phosphate buffer pH 7.4 in D_2_O, giving a 700 µL sample containing 4 mM TSP and 53.85 µM (31.25 µg/mL) MA as an internal standard for quantification. Finally, the samples were transferred into 5 mm NMR tubes and immediately analyzed.

### 4.5. Metabolite Spiking

Stock solutions for metabolite spiking were prepared by dissolving 1 mg of a known metabolite in 100 µL reference plasma. In the next step, 10 to 30 µL of this stock solution was added to a standard NMR sample (350 µL reference plasma and 350 µL buffer containing TSP and MA), creating a spiked plasma sample for each metabolite separately. Each sample was subsequently analyzed by ^1^H-NMR spectroscopy using the experimental parameters as described above. This procedure was repeated for 62 metabolites (see Table 1).

### 4.6. ^1^H-NMR Analysis

*NMR data acquisition.* After thermal sample stabilization for 5 min at 25 °C, ^1^H-NMR spectra were recorded at 25 °C with 96 scans (total measurement time of 9 min) on a 600 MHz JEOL NMR spectrometer having a magnetic field strength of 14.1 Tesla. Slightly T_2_-weighted spectra were acquired using the CPMG pulse sequence to attenuate signals of remaining plasma proteins, such as albumins with a short T_2_ relaxation time. The CPMG pulse sequence (total spin-echo time of 64 ms; spin-echo delay of 0.4 ms; 160 loops) was preceded by 16 prescans. Water suppression was accomplished by presaturation for 3 s. Other parameters used were as follows: 16 k data points, a spectral width of 12 ppm, and an acquisition time of 2.27 s.

*NMR spectra processing*. Spectra were processed using JEOL Delta software (version 5.3.1) for all following processing steps. A line broadening of 0.8 Hz and a zero-filling factor of four to 64 k datapoints was applied. After Fourier transformation, the spectra were phased manually and baseline corrected. The upfield peak of the methyl doublet of alanine was used to calibrate the ppm chemical shift scale at 1.4938 ppm. Finally, spectra were divided into 237 fixed integration regions, rationally defined based on the metabolite spiking results. For the TSP–HSA binding experiments described in Figure 1A,B, non-IS-normalized integration values (area under the peaks) were used, i.e., integration values not normalized against an internal standard (IS, of known and fixed concentration), but normalized against a freely chosen, but fixed, normalization value. This approach can be used since spectra without TSP and with 4 mM TSP are taken immediately after each other under fully identical NMR measuring conditions (10 µL of TSP-containing buffer solution was added to the NMR tube after the first measurement). For the orthogonal partial least squares discriminant analysis (OPLS-DA) model training and validation, all integration values of the 237 integration regions were normalized to the integration value of the MA internal standard.

### 4.7. Statistical Analysis for Model Training and Valorization

Multivariate statistical analysis was performed using SIMCA^®^ (version 15.0.2). All variables were normalized to the integration value of MA, mean-centered, and Pareto scaled. Variables showing high variation, i.e., an intrasample %RSD >10% (9 variables) or an intersample %RSD >30% (7 variables), were removed from the dataset. The first training model was constructed using a large cohort of 80 lung cancer patients and 80 healthy controls, and all remaining 221 variables by means of OPLS-DA. In the next step, data reduction was performed by removing ‘noisy’ variables that, based on their loading values and their standard errors calculated by cross-validation (jack-knifing), showed little or no significant contribution to the model (143 variables). More specifically, a variable is considered significant if its loading (absolute) value exceeds its standard error defined by cross-validation. Interpretation of variable significance by comparing its loading and its standard error resulting from cross-validation (jack-knife interval) is a commonly used method for variable selection, by taking the error on the predicted scores from all cross-validation rounds into account [76,77,78,79,80,81]. Using this reduced dataset of 78 variables (221 minus 143 variables), an unsupervised principal component analysis (PCA) model was constructed to confirm the separation of the two groups. Moreover, a receiver operating characteristic (ROC) curve was constructed to evaluate the selection of significant variables, where an AUC (area under the curve) value close to 1 indicates a strong model. The same dataset was also used to construct the final supervised OPLS-DA classifier (trained classification model). Finally, the classifier was validated by (i) a seven-fold internal cross-validation, (ii) a permutation testing, where an R^2^ value of ±0.2 and a negative Q^2^ value typically indicate a good model, and (iii) a validation using an independent validation cohort of 34 lung cancer patients and 38 controls.

### 4.8. Metabolite Identification

From the 78 variables that were used to construct the discriminative OPLS-DA model, 30 variables with a VIP (variable importance in projection) value >0.80 were selected. An S-plot was used to identify which variables/metabolites are increased or decreased in lung cancer patients compared to healthy controls. ^1^H-NMR spectra of individuals with high and low values for these variables were selected and the corresponding peaks and their J-coupling multiplicities were compared to ensure correct metabolite identification for the variables that show strong discriminative power between the two groups.

## 5. Conclusions

This paper validates the use of maleic acid (MA) as an internal standard to quantify the human plasma metabolite profile with ^1^H-NMR spectroscopy and to detect metabolic changes occurring in patients with diseases, as demonstrated in this work for lung cancer. It is shown that by adding 4 mM TSP as a strong competitor, the metabolite peak intensities become independent of the varying sample-to-sample human serum albumin (HSA) concentration, thus avoiding the need for (low-reproducible) protein precipitation. Based on metabolite spiking, and using the methyl signal of alanine to calibrate the ppm chemical shift scale, the plasma ^1^H-NMR spectrum is divided into 237 fixed integration regions, serving as variables in multivariate statistics. The resulting classification model allows discrimination between 80 lung cancer patients and 80 healthy controls, with a specificity of 93% and a sensitivity of 85%, in combination with an area under the curve of 0.95. Last, but not least, the robustness of the classifier is demonstrated in an independent validation cohort (*n* = 72).

## Figures and Tables

**Table 1:** Summary of the 62 spiked metabolites and their chemical shifts, multiplicity, and J-coupling. The assigned integration numbers for each proton are listed, and can be linked with the integration regions shown in Table S1. d: doublet; dd: double doublet; ddd: doublet of double doublet; dq: double quadruplet; dt: double triplet; m: multiplet; p: pentaplet; q: quadruplet; s: singlet; t: triplet.

Metabolite	Proton	Chemical Shift (ppm)	Multiplicity and J-Coupling (Hz)	Connectivity	Assigned Integration Number (VAR)
1-methylhistidine 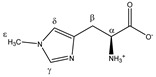	^α^CH	3.959	dd (7.8; 5.6)	α–β; α–β’	051–054
	^β^CH_2_	3.226	dd_(1)_ (16.1; 7.8)	β–β’; β–α	123, 125, 128, 130
		3.315	dd_(2)_ (16.1; 5.6)	β’–β; β’–α	113–115, 117
	^γ^CH	7.917	s	/	004
	^δ^CH	7.064	s	/	017
	^ε^CH_3_	3.723	s	/	076
2-aminobutyrate 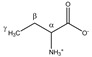	^α^CH	3.736	t (5.9)	α–β; α–β’	074–076
	^β^CH_2_	1.920	m	/	197–200
	^γ^CH_3_	0.999	t (7.5)	γ–β; γ–β’	228, 230, 231
2-hydroxybutyrate 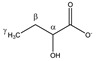	^α^CH	4.017	dd (6.0; 4.5)	α–β; α–β’	045, 046
	^β^CH_2_	1.762	m	/	202, 203
		1.670	p	/	204, 205, 207
	^γ^CH_3_	0.920	t (7.5)	γ–β; γ–β’	235, 236
2-hydroxy- 3-methylbutyrate 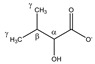	^α^CH	3.866	d	α–β	063
	^β^CH_2_	2.034	m	/	194–195
	^γ^CH_3_	0.986	d_(1)_	γ–β	231–232
		0.853	d_(2)_	γ’–β	237
3-hydroxy- 3-methylbutyrate 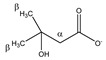	^α^CH_2_	2.383	s	/	177
	^β^CH_3_	1.287	s	/	218
3-methyl- 2-oxobutyrate 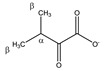	^α^CH	3.049	m	/	138–143, 145
	^β^CH_3_	1.139	d (6.8)	β–α	223
3-methyl- 2-oxovalerate 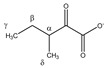	^α^CH	2.947	m	/	149, 150, 152
	^β^CH_2_	1.721	m_(1)_	/	202–204
		1.463	m_(2)_	/	212, 213
	^γ^CH_3_	0.907	t (7.0)	γ–β; γ–β’	236
	^δ^CH_3_	1.113	d (6.8)	δ–α	224
3-methylhistidine 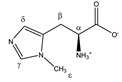	^α^CH	3.986	dd (7.9; 4.8)	α–β; α–β’	048–051
	^β^CH_2_	3.091	dd_(1)_ (15.5; 7.9)	β–β’; β–α	136–139
		3.189	dd_(2)_ (15.5; 4.8)	β’–β; β’–α	130, 132, 133
	^γ^CH	7.679	s	/	009
	^δ^CH	7.025	s	/	017
	^ε^CH_3_	3.711	s	/	077
4-aminobutyrate 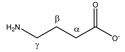	^α^CH_2_	2.316	t (7.4)	α–β	180, 181, 183
	^β^CH_2_	1.922	p	/	199, 200
	^γ^CH_2_	3.033	t (7.4)	γ–β	142, 144, 146
4-methyl- 2-oxovalerate 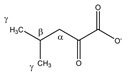	^α^CH_2_	2.625	d (6.5)	α–β	166
	^β^CH	2.112	m	/	190–192
	^γ^CH_3_	0.948	d (6.5)	γ–β	234
α-ketoglutarate 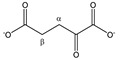	^α^CH_2_	3.030	t (6.9)	α–β	143, 145, 147
	^β^CH_2_	2.461	t (6.9)	β–α	172, 173
Acetate * 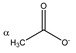 *	^α^CH_3_	1.938	s	/	199
Acetoacetate 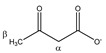	^α^CH_2_	3.464	s	/	101
	^β^CH_3_	2.298	s	/	183
Alanine 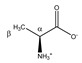	^α^CH	3.805	q (7.2)	α–β	067–070
	^β^CH_3_	1.500	d (7.2)	β–α	211
Allantoin 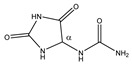	^α^CH	5.410	s	/	024
Arginine 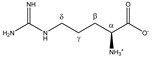	^α^CH	3.791	t (6.1)	α–β; α–β’	069–071
	^β^CH_2_	1.938	m	/	197–200
	^γ^CH_2_	1.751	m_(1)_	/	202–204
		1.672	m_(2)_	/	204–207
	^δ^CH_2_	3.265	t (6.9)	δ–γ; δ–γ’	120–122
Asparagine 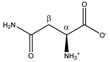	^α^CH	4.020	dd (7.7; 4.4)	α–β; α–β’	045, 046
	^β^CH_2_	2.877	dd_(1)_ (16.8; 7.7)	β–β’; β–α	153, 154
		2.970	dd_(2)_ (16.8; 4.4)	β’–β; β’–α	148, 150, 151
Aspartate 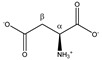	^α^CH	3.921	dd (8.8; 3.7)	α–β; α–β’	056, 057, 058
	^β^CH_2_	2.699	dd_(1)_ (17.5; 8.8)	β–β’; β–α	160, 162–164
		2.833	dd_(2)_ (17.5; 3.7)	β’–β; β’–α	155
β-alanine 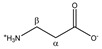	^α^CH_2_	2.575	t (6.8)	α–β	167–168
	^β^CH_2_	3.201	t (6.8)	β–α	129–131
β-hydroxybutyrate 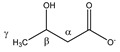	^α^CH_2_	2.426	dd_(1)_ (14.4; 7.4)	α–α’; α–β	173–176
		2.325	dd_(2)_ (14.4; 6.4)	α’–α; α’–β	179–182
	^β^CH	4.172	m	/	037, 038, 040
	^γ^CH_3_	1.219	d (6.3)	γ–β	220
Betaine 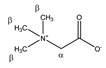	^α^CH_2_	3.921	s	/	057
	^β^CH_3_	3.285	s	/	118
Carnitine 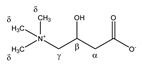	^α^CH_2_	2.479	dd_(1)_ (15.4; 7.1)	α–α’; α–β	170, 172
		2.439	dd_(2)_ (15.4; 6.4)	α’–α; α’–β	172–175
	^β^CH	4.588	m	/	029
	^γ^CH_2_	3.450	m	/	102, 103
	^δ^CH_3_	3.246	s	/	123
Choline 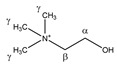	^α^CH_2_	4.086	m	/	042
	^β^CH_2_	3.539	m	/	094–096
	^γ^CH_3_	3.222	s	/	128
Citrate 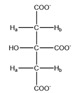	CH_2a_	2.693	d (15.6)	a–b	162,164
	CH_2b_	2.553	d (15.6)	b–a	168
Creatine 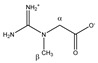	^α^CH_2_	3.950	s	/	054
	^β^CH_3_	3.056	s	/	141
Creatinine 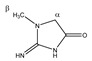	^α^CH_2_	4.074	s	/	042
	^β^CH_3_	3.063	s	/	140
Cysteine 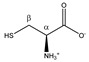	^α^CH	3.979	dd (5.8; 4.1)	α–β; α–β’	049–051
	^β^CH_2_	3.112	dd_(1)_ (14.8; 5.8)	β–β’; β–α	135–137
		3.052	dd_(2)_ (14.8; 4.1)	β’–β; β’–α	139, 140, 142, 143
Cystine 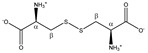	^α^CH	4.126	dd (8.2; 4.1)	α–β; α–β’	040, 041
	^β^CH_2_	3.210	dd_(1)_ (14.8; 8.2)	β–β’; β–α	126, 129–131
		3.405	dd_(2)_ (14.8; 4.1)	β’–β; β’–α	106, 108
Formate 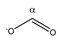	^α^CH	8.477	s	/	001
Fumarate * 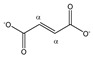 *	^α^CH	6.540	s	/	021
Glucose α-anomer 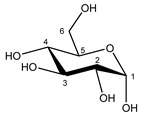 β-anomer * 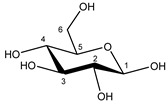 *	C1H	5.256	d (3.8)	/	025
	C2H	3.557	dd (9.8; 3.8)	/	093, 094
	C3H	3.736	t (9.6)	/	073–076
	C4H	3.435	t (9.6)	/	103, 104, 106
	C5H	3.857	m	/	062–066
	C6H	3.854	dd_(1)_ (12.2; 7.8)	/	062, 063, 065, 066
	C6′H	3.788	dd_(2)_ (12.2; 5.4)	/	069–071
				
	C1H	4.669	d (7.8)	/	028
	C2H	3.267	dd (9.4; 8.0)	/	119, 121, 122
	C3H	3.513	t (9.2)	/	096, 097, 099
	C4H	3.425	t (9.4)	/	103, 106, 107
	C5H	3.487	m	/	098–100
	C6H	3.747	dd_(1)_ (12.2; 5.8)	/	073–075
	C6′H	3.920	dd_(2)_ (12.2; 2.0)	/	056–059
Glutamate 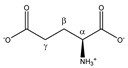	^α^CH	3.779	dd (7.3; 4.7)	α–β; α–β’	070–072
	^β^CH_2_	2.152	m_(1)_	/	189–191
		2.075	m_(2)_	/	192–194
	^γ^CH_2_	2.372	m	/	176–180
Glutamine 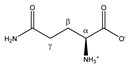	^α^CH	3.793	t (6.1)	α–β; α–β’	069–071
	^β^CH_2_	2.157	m	/	189–192
	^γ^CH_2_	2.474	m	/	169–174
Glycerol 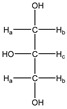	CH_2a_	3.582	dd (11.8; 6.5)	a–b; a–c	090–093
	CH_2b_	3.674	dd (11.8; 4.3)	b–a; b–c	079, 080, 082, 083
	CH_c_	3.806	m	/	067–070
Glycine 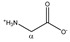	^α^CH_2_	3.580	s	/	092
Histidine 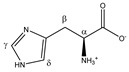	^α^CH	4.007	dd (7.9; 4.9)	α–β; α–β’	046–048
	^β^CH_2_	3.160	dd_(1)_ (15.5; 7.9)	β–β’; β–α	132, 134
		3.262	dd_(2)_ (15.5; 4.9)	β’–β; β’–α	119, 120, 122, 123
	^γ^CH	7.864	s	/	006
	^δ^CH	7.101	s	/	016
Hydroxyproline 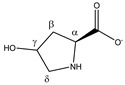	^α^CH	4.370	dd (10.3; 8.1)	α–β; α–β’	033
	^β^CH_2_	2.180	ddd (13.8; 10.3; 4.2)	β–β’; β–α; β–γ	189
		2.450	ddt (13.8; 8.1; 1.7; 1.7)	β’–β; β’–α; β’–γ; β’–δ’	172–174
	^γ^CH	4.690	m	/	028
	^δ^CH_2_	3.510	dd (12.6; 3.4)	δ–δ’; δ–γ	097, 099
		3.391	dt (12.6; 1.7; 1.7)	δ’–δ; δ’–γ; δ’–β’	108–110
Hypoxanthine 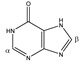	^α^CH	8.222	s	/	002
	^β^CH	8.203	s	/	002
Isoleucine 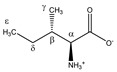	^α^CH	3.693	d (4.0)	α–β	079
	^β^CH	2.002	m	/	195–197
	^γ^CH_3_	1.030	d (7.0)	γ–β	227
	^δ^CH_2_	1.492	m_(1)_	/	210–212
		1.282	m_(2)_	/	217–220
	^ε^CH_3_	0.958	t (7.4)	ε–δ; ε–δ’	233, 234
Isopropanol 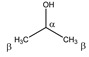	^α^CH	4.039	m	/	042–046
	^β^CH_3_	1.191	d (6.2)	β–α	222
Lactate 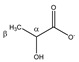	^α^CH	4.133	q (6.9)	α–β	039–041
	^β^CH_3_	1.348	d (6.9)	β–α	215
Leucine 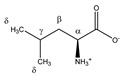	^α^CH	3.756	dd (8.6; 4.9)	α–β; α–β’	072–074
	^β^CH_2_	1.738	m	/	202–204
	^γ^CH	1.738	m	/	202–204
	^δ^CH_3_	0.986	d_(1)_ (6.3)	δ–γ	231, 232
		0.975	d_(2)_ (6.3)	δ’–γ	232, 233
Lysine 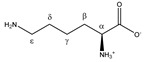	^α^CH	3.777	t (6.1)	α–β; α–β’	070–072
	^β^CH_2_	1.926	m	/	198–200
	^γ^CH_2_	1.534	m_(1)_	/	209–211
		1.463	m_(2)_	/	211–213
	^δ^CH_2_	1.747	p	/	202, 203
	^ε^CH_2_	3.046	t (7.6)	ε–δ	141, 142, 144
Mannose α-anomer 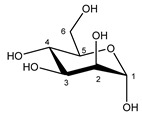 β-anomer * 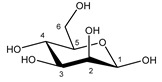 *	C1H	5.205	d (1.8)	/	026
	C2H	3.957	dd (3.4; 1.8)	/	052–054
	C3H	3.870	dd (9.7; 3.6)	/	062, 063
	C4H	3.683	t (9.7)	/	079, 080, 082
	C5H	3.837	m	/	064–067
	C6H	3.787	dd_(1)_ (12.2; 5.5)	/	069–071
	C6′H	3.895	dd_(2)_ (12.2; 2.3)	/	059, 061, 062
	C1H	4.924	d (1.2)	/	027
	C2H	3.968	dd (3.3; 1.2)	/	051, 052
	C3H	3.681	dd (9.7; 3.3)	/	079, 081, 082
	C4H	3.598	t (9.7)	/	088, 090, 092
	C5H	3.404	m	/	106–108
	C6H	3.757	dd_(1)_ (12.2; 6.4)	/	071, 073, 074
	C6′H	3.928	dd_(2)_ (12.2; 2.3)	/	055, 057
Methionine 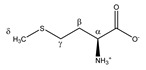	^α^CH	3.878	dd (7.1; 5.5)	α–β; α–β’	061–063
	^β^CH_2_	2.220	m_(1)_	/	187–188
		2.142	m_(2)_	/	190–192
	^γ^CH_2_	2.664	t (7.6)	γ–β; γ–β’	164, 165
	^δ^CH_3_	2.156	s	/	189
Myo-inositol 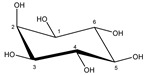	C1H	3.645	t (9.8)	/	083, 085, 087
	C2H	3.302	t (9.4)	/	114, 116, 118
	C3H	3.645	t (9.8)	/	083, 085, 087
	C4H	3.557	dd (9.8; 2.9)	/	093, 094
	C5H	4.085	t (2.9)	/	042
	C6H	3.557	dd (9.8; 2.9)	/	093, 094
N-acetylcysteine 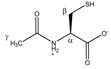	^α^CH	4.407	m	/	033
	^β^CH_2_	2.946	m	/	149, 151, 152
	^γ^CH_3_	2.090	s	/	193
Ornithine 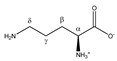	^α^CH	3.802	t (5.9)	α–β	068–070
	^β^CH_2_	1.966	m	/	196–199
	^γ^CH_2_	1.855	m_(1)_	/	200, 201
		1.772	m_(2)_	/	201, 202
	^δ^CH_2_	3.075	t (7.6)	δ–y; δ–γ’	138–140
Oxaloacetate 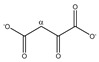	^α^CH_2_	2.390	s	/	177
Phenylalanine 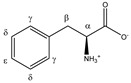	^α^CH	4.014	dd (7.8; 5.3)	α–β; α–β’	045–047
	^β^CH_2_	3.303	dd_(1)_ (14.5; 7.8)	β–β’; β–α	114, 115, 117, 118
		3.147	dd_(2)_ (14.5; 5.3)	β’–β; β’–α	134, 135
	^γ^CH	7.349	d (7.3)	γ–δ	011, 012
	^δ^CH	7.446	t (7.3)	δ–ε; δ–γ	011
	^ε^CH	7.392	t (7.3)	ε–δ	011
Proline 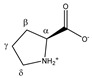	^α^CH	4.152	dd (8.9; 6.4)	α–β; α–β’	038–040
	^β^CH_2_	2.371	m_(1)_	/	177–179
		2.091	m_(2)_	/	191–194
	^γ^CH_2_	2.024	m	/	194–197
	^δ^CH_2_	3.441	dt_(1)_ (11.6; 7.1; 2.6)	δ–δ’/δ–γ/δ–β’	101, 103, 105, 106
		3.359	dt_(2)_ (11.6; 7.1; 2.6)	δ’–δ/δ’–γ/δ’–β’	110–113
Pyroglutamate 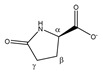	^α^CH	4.196	dd (8.6; 6.2)	α–β; α–β’	037
	^β^CH_2_	2.526	m_(1)_	/	168–170
		2.055	m_(2)_	/	193–195
	^γ^CH_2_	2.242	m	/	174–176
Pyruvate 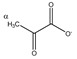	^α^CH_3_	2.390	s	/	177
Sarcosine 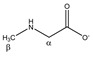	^α^CH_2_	3.632	s	/	087
	^β^CH_3_	2.759	s	/	157
Serine 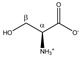	^α^CH	3.863	dd (5.7; 3.7)	α–β; α–β’	063, 064
	^β^CH_2_	3.968	dd_(1)_ (12.2; 5.7)	β–β’; β–α	050–052, 054
		4.011	dd_(2)_ (12.2; 3.7)	β’–β; β’–α	045–048
Succinate 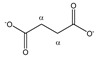	^α^CH_2_	2.424	s	/	175
Taurine 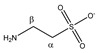	^α^CH_2_	3.282	t (6.6)	α–β	117, 119, 120
	^β^CH_2_	3.442	t (6.6)	β–α	103, 105
Threonine 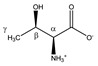	^α^CH	3.606	d (4.9)	α–β	088, 089
	^β^CH	4.275	dq (6.6; 4.9)	β–γ; β–α	035, 036
	^γ^CH_3_	1.349	d (6.6)	γ–β	215
Tryptophan 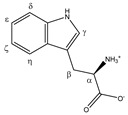	^α^CH	4.078	dd (7.7; 5.1)	α–β; α–β’	042
	^β^CH_2_	3.329	dd_(1)_ (15.4; 7.7)	β–β’; β–α	112–115
		3.502	dd_(2)_ (15.4; 5.1)	β’–β; β’–α	097–100
	^γ^CH	7.344	s	/	012
	^δ^CH	7.552	d (7.8)	δ–ε	010
	^ε^CH	7.207	t (7.8)	ε–δ; ε–ζ	015
	^ζ^CH	7.288	t (7.8)	ζ–ε; ζ–η	013
	^η^CH	7.750	d (7.8)	η–ζ	008
Tyrosine 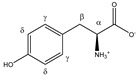	^α^CH	3.960	dd (7.7; 5.2)	α–β; α–β’	051–053
	^β^CH_2_	3.075	dd_(1)_ (14.5; 7.7)	β–β’; β–α	137–139, 141
		3.218	dd_(2)_ (14.5; 5.2)	β’–β; β’–α	125, 127–130
	^γ^CH	6.918	d (8.4)	γ–δ	019
	^δ^CH	7.212	d (8.4)	δ–γ	015
Uridine 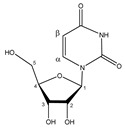	^α^CH	7.892	d (8.1)	α–β	005
	^β^CH	5.921	d (8.1)	β–α	022
	C1H	5.937	d (4.6)	/	022
	C2H	4.375	t (4.9)	/	033
	C3H	4.250	t (5.4)	/	036
	C4H	4.152	m	/	038–040
	C5H	3.828	dd_(1)_ (12.3; 4.7)	/	066–068
	C5′H	3.930	dd_(2)_ (12.3; 3.0)	/	055, 057
Valine 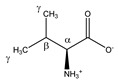	^α^CH	3.632	d (4.3)	α–β	086, 087
	^β^CH	2.294	m	/	180–185
	^γ^CH_3_	1.062	d_(1)_ (7.1)	γ–β	226
		1.010	d_(2)_ (7.1)	γ’–β	228, 229

**Table 2:** Clinical characteristics of the subjects included in the training and validation cohort. BMI: body mass index; COPD: chronic obstructive pulmonary disease; LC: lung cancer; NOS: not otherwise specified; NSCLC: non-small-cell lung cancer; SCLC: small-cell lung cancer. Note: clinical tumor staging in the NCT02024113 study was performed according to TNM classification, 7th edition.

		Training Cohort		Validation Cohort	
		Controls	LC Patients	Controls	LC Patients
Number of patients, *n*		80	80	38	34
Sex, *n* (%)	Male	48 (60)	54 (68)	19 (50)	24 (71)
	Female	32 (40)	26 (33)	19 (50)	10 (29)
Age, years (range)		67 ± 10 (46–85)	68 ± 10 (43–88)	68 ± 12 (38–88)	70 ± 10 (36–83)
BMI, kg/m^2^ (range)		28.8 ± 5.5 (18.7–46.7)	26.0 ± 4.3 (18.4–38.5)	29.8 ± 5.0 (20.8–46.6)	25.7 ± 5.0 (19.9–41.4)
Smoking status, *n* (%)	Active smoker	16 (20)	34 (43)	5 (13)	19 (56)
	Ex-smoker (>6 months)	38 (48)	40 (50)	18 (47)	14 (41)
	Non-smoker	26 (33)	6 (8)	15 (39)	1 (3)
Packyears, years (range)		13 ± 19 (0–94)	35 ± 22 (0–125)	15 ± 25 (0–125)	40 ± 22 (0–90)
COPD, *n* (%)		5 (6)	35 (44)	7 (18)	17 (50)
Diabetes, *n* (%)		17 (21)	16 (20)	11 (29)	5 (15)
Number of tumors, *n*			85		34
Tumor histology, *n* (%)	NSCLC, adenocarcinoma		27 (32)		10 (29)
	NSCLC, squamous carcinoma		26 (31)		8 (24)
	NSCLC, adenosquamous carcinoma		2 (2)		0 (0)
	NSCLC, carcinoid		1 (1)		1 (3)
	NSCLC, NOS		3 (4)		2 (6)
	SCLC		11 (13)		8 (24)
	Unknown		15 (18)		5 (15)
Tumor stage, *n* (%)	IA		17 (20)		10 (29)
	IB		6 (7)		1 (3)
	IIA		7 (8)		0 (0)
	IIB		6 (7)		1 (3)
	IIIA		21 (25)		9 (26)
	IIIB		9 (11)		5 (15)
	IV		19 (22)		8 (24)

## Data Availability

The data presented in this study are available on request.

## Supplementary Materials

The following are available online at https://www.mdpi.com/article/10.3390/metabo11080537/s1, Figure S1: zoom-in of a lactate signal upon the addition of different amounts of TSP, Figure S2: OPLS-DA classifier of the training cohort using 221 variables, Figure S3: principal component analysis (PCA) of all 232 subjects from the training and validation cohort, Figure S4: permutation test of the training model, Table S1: overview of the 237 integration numbers (variables), and their corresponding integration regions and contributing metabolites, Table S2: overview of the intrasample variability of the 237 integration regions, Table S3: overview of the 30 variables with the highest total VIP value based on the OPLS-DA model that was constructed with 78 variables.
