# Supplementary material for: Detection of Lung Cancer via Blood Plasma and 1H-NMR Metabolomics: Validation by a Semi-Targeted and Quantitative Approach Using a Protein-Binding Competitor
Source: Metabolites. 2021 Aug 12;11(8):537. doi: 10.3390/metabo11080537 (PMC8401204; doi:10.3390/metabo11080537)
Supplement: Supplementary file 1 [file metabolites-11-00537-s001.zip › metabolites-1285618-supplementary.pdf]

Table S1. Overview of the 237 integration numbers (variables) and their corresponding integration regions and contributing metabolites. All integration regions and corresponding metabolites are defined by spiking experiments. FAC: fatty acid chain; NAG: N-acetylated glycoproteins; NI: non-identified; PC: phosphatidylcholine; PL: phospholipids; SM: sphingomyelin; TG: triglycerides; VAR: variable (assigned integration number).

Assigned integration number (variable)	Integration region (ppm)	Corresponding metabolite(s)
**VAR 001**	**8.460 - 8.484**	Formate
**VAR 002**	**8.190 - 8.240**	Hypoxanthine
**VAR 003**	**7.945 - 8.170**	NI 1
**VAR 004**	**7.905 - 7.945**	1-methylhistidine
**VAR 005**	**7.875 - 7.905**	Uridine
**VAR 006**	**7.845 - 7.875**	Histidine
**VAR 007**	**7.820 - 7.845**	NI 2
**VAR 008**	**7.720 - 7.780**	Tryptophan<br>NI 3
**VAR 009**	**7.650 - 7.700**	3-methylhistidine
**VAR 010**	**7.530 - 7.580**	Tryptophan
**VAR 011**	**7.350 - 7.480**	Phenylalanine
**VAR 012**	**7.330 - 7.350**	Phenylalanine<br>Tryptophan
**VAR 013**	**7.260 - 7.320**	Tryptophan<br>NI 4
**VAR 014**	**7.240 - 7.260**	NI 5
**VAR 015**	**7.180 - 7.240**	Tryptophan<br>Tyrosine
**VAR 016**	**7.080 - 7.140**	Histidine
**VAR 017**	**6.998 - 7.071**	1-methylhistidine<br>3-methylhistidine<br>NI 6
**VAR 018**	**6.954 - 6.986**	NI 7
**VAR 019**	**6.890 - 6.950**	Tyrosine
**VAR 020**	**6.730 - 6.775**	NI 8
**VAR 021**	**6.533 - 6.547**	Fumarate
**VAR 022**	**5.907 - 5.946**	Uridine
**VAR 023**	**5.720 - 5.865**	NI 9
**VAR 024**	**5.405 - 5.415**	Allantoin
**VAR 025**	**5.238 - 5.267**	Glucose
**VAR 026**	**5.196 - 5.210**	Mannose
**VAR 027**	**4.919 - 4.928**	Mannose
**VAR 028**	**4.633 - 4.703**	Glucose<br>Hydroxyproline<br>NI 10
**VAR 029**	**4.560 - 4.614**	Carnitine
**VAR 030**	**4.528 - 4.540**	NI 11
**VAR 031**	**4.521 - 4.527**	NI 12
**VAR 032**	**4.450 - 4.490**	NI 13

**VAR 033**	**4.347 - 4.430**	Hydroxyproline<br>N-acetylcysteine<br>Uridine<br>Lipids: C_1H and C_3H in glycerol backbone of PL and TG
**VAR 034**	**4.299 - 4.347**	Lipids: (O-C**H₂**-CH₂-N⁺(CH₃)₃ of PC and SM; C_1H and C_3H in glycerol backbone of PL and TG
**VAR 035**	**4.269 - 4.299**	Threonine<br>Lipids: (O-C**H₂**-CH₂-N⁺(CH₃)₃ of PC and SM
**VAR 036**	**4.237 - 4.269**	Threonine<br>Uridine
**VAR 037**	**4.172 - 4.216**	Pyroglutamate<br>β-Hydroxybutyrate
**VAR 038**	**4.153 - 4.172**	Proline<br>Uridine<br>β-Hydroxybutyrate
**VAR 039**	**4.147 - 4.153**	Lactate<br>Proline<br>Uridine
**VAR 040**	**4.133 - 4.147**	Lactate<br>Proline<br>Uridine<br>β-Hydroxybutyrate<br>Cystine
**VAR 041**	**4.110 - 4.133**	Lactate<br>Cystine
**VAR 042**	**4.054 - 4.102**	Choline<br>Creatinine<br>Myoinositol<br>Tryptophan<br>Isopropanol<br>NI 14
**VAR 043**	**4.043 - 4.054**	Isopropanol<br>NI 14
**VAR 044**	**4.034 - 4.043**	Isopropanol
**VAR 045**	**4.021 - 4.034**	2-hydroxybutyrate<br>Asparagine<br>Phenylalanine<br>Serine<br>Isopropanol
**VAR 046**	**4.006 - 4.021**	2-hydroxybutyrate<br>Asparagine<br>Histidine<br>Phenylalanine<br>Serine<br>Isopropanol
**VAR 047**	**4.001 - 4.006**	Histidine<br>Phenylalanine

		Serine
**VAR 048**	**3.993 - 4.001**	3-methylhistidine<br>Histidine<br>Serine
**VAR 049**	**3.985 - 3.992**	3-methylhistidine<br>Cysteine
**VAR 050**	**3.979 - 3.985**	3-methylhistidine<br>Cysteine<br>Serine
**VAR 051**	**3.968 - 3.979**	1-methylhistidine<br>3-methylhistidine<br>Cysteine<br>Mannose<br>Serine<br>Tyrosine
**VAR 052**	**3.960 - 3.968**	1-methylhistidine<br>Mannose<br>Serine<br>Tyrosine
**VAR 053**	**3.954 - 3.960**	1-methylhistidine<br>Mannose<br>Tyrosine
**VAR 054**	**3.946 - 3.954**	1-methylhistidine<br>Creatine<br>Mannose<br>Serine
**VAR 055**	**3.934 - 3.946**	Mannose<br>Uridine
**VAR 056**	**3.930 - 3.934**	Aspartate<br>Glucose
**VAR 057**	**3.914 - 3.930**	Aspartate<br>Betaine<br>Glucose<br>Mannose<br>Uridine
**VAR 058**	**3.909 - 3.914**	Aspartate<br>Glucose
**VAR 059**	**3.900 - 3.909**	Glucose<br>Mannose<br>NI 15
**VAR 060**	**3.893 - 3.900**	NI 15
**VAR 061**	**3.885 - 3.893**	Mannose<br>Methionine
**VAR 062**	**3.873 - 3.885**	Glucose<br>Mannose<br>Methionine<br>NI 15

**VAR 063**	**3.857 - 3.873**	2-hydroxy-3-methylbutyrate<br>Glucose<br>Mannose<br>Methionine<br>Serine
**VAR 064**	**3.851 - 3.857**	Glucose<br>Mannose<br>Serine
**VAR 065**	**3.846 - 3.851**	Glucose<br>Mannose
**VAR 066**	**3.829 - 3.844**	Glucose<br>Mannose<br>Uridine
**VAR 067**	**3.818 - 3.829**	Alanine<br>Mannose<br>Uridine<br>Glycerol
**VAR 068**	**3.808 - 3.818**	Alanine<br>Ornithine<br>Uridine<br>Glycerol
**VAR 069**	**3.796 - 3.808**	Alanine<br>Arginine<br>Glucose<br>Glutamine<br>Mannose<br>Ornithine<br>Glycerol
**VAR 070**	**3.785 - 3.796**	Alanine<br>Arginine<br>Glucose<br>Glutamate<br>Glutamine<br>Lysine<br>Mannose<br>Ornithine<br>Glycerol
**VAR 071**	**3.770 - 3.785**	Arginine<br>Glucose<br>Glutamate<br>Glutamine<br>Lysine<br>Mannose
**VAR 072**	**3.765 - 3.770**	Glutamate<br>Leucine<br>Lysine
**VAR 073**	**3.748 - 3.765**	Glucose

		Leucine<br>Mannose
**VAR 074**	**3.739 - 3.748**	Glucose<br>Leucine<br>Mannose<br>2-aminobutyrate
**VAR 075**	**3.728 - 3.739**	Glucose<br>2-aminobutyrate
**VAR 076**	**3.715 - 3.728**	1-methylhistidine<br>Glucose<br>2-aminobutyrate<br>NI 16
**VAR 077**	**3.709 - 3.713**	3-methylhistidine
**VAR 078**	**3.704 - 3.709**	NI 16
**VAR 079**	**3.685 - 3.704**	Isoleucine<br>Mannose<br>Glycerol<br>NI 16<br>NI 17<br>Lipids: (O-CH_2-C**H_2**-$N^+(CH_3)_3$ of PC and SM
**VAR 080**	**3.678 - 3.685**	Mannose<br>Glycerol<br>NI 17<br>Lipids: (O-CH_2-C**H_2**-$N^+(CH_3)_3$ of PC and SM
**VAR 081**	**3.673 - 3.678**	Mannose<br>Lipids: (O-CH_2-C**H_2**-$N^+(CH_3)_3$ of PC and SM
**VAR 082**	**3.664 - 3.673**	Mannose<br>Glycerol<br>NI 17<br>Lipids: (O-CH_2-C**H_2**-$N^+(CH_3)_3$ of PC and SM
**VAR 083**	**3.654 - 3.664**	Myoinositol<br>Glycerol<br>NI 17<br>Lipids: (O-CH_2-C**H_2**-$N^+(CH_3)_3$ of PC and SM
**VAR 084**	**3.649 - 3.654**	Lipids: (O-CH_2-C**H_2**-$N^+(CH_3)_3$ of PC and SM
**VAR 085**	**3.642 - 3.647**	Myoinositol
**VAR 086**	**3.633 - 3.641**	Valine
**VAR 087**	**3.623 - 3.633**	Myoinositol<br>Sarcosine<br>Valine
**VAR 088**	**3.608 - 3.616**	Mannose<br>Threonine
**VAR 089**	**3.600 - 3.606**	Threonine
**VAR 090**	**3.594 - 3.600**	Mannose<br>Glycerol
**VAR 091**	**3.584 - 3.589**	Glycerol
**VAR 092**	**3.576 - 3.584**	Glycine

		Mannose<br>Glycerol
**VAR 093**	**3.559 - 3.572**	Glucose<br>Myoinositol<br>Glycerol
**VAR 094**	**3.541 - 3.555**	Choline<br>Glucose<br>Myoinositol
**VAR 095**	**3.534 - 3.541**	Choline
**VAR 096**	**3.524 - 3.534**	Choline<br>Glucose
**VAR 097**	**3.507 - 3.522**	Glucose<br>Hydroxyproline<br>Tryptophan
**VAR 098**	**3.500 - 3.504**	Glucose<br>Tryptophan
**VAR 099**	**3.490 - 3.500**	Glucose<br>Hydroxyproline<br>Tryptophan
**VAR 100**	**3.467 - 3.490**	Glucose<br>Tryptophan
**VAR 101**	**3.460 - 3.467**	Proline<br>Acetoacetate
**VAR 102**	**3.455 - 3.460**	Carnitine
**VAR 103**	**3.437 - 3.455**	Carnitine<br>Glucose<br>Proline<br>Taurine
**VAR 104**	**3.433 - 3.437**	Glucose
**VAR 105**	**3.429 - 3.433**	Proline<br>Taurine
**VAR 106**	**3.413 - 3.429**	Mannose<br>Glucose<br>Proline<br>Cystine
**VAR 107**	**3.401 - 3.413**	Glucose<br>Mannose
**VAR 108**	**3.387 - 3.401**	Hydroxyproline<br>Mannose<br>Cystine
**VAR 109**	**3.383 - 3.387**	Hydroxyproline
**VAR 110**	**3.375 - 3.383**	Hydroxyproline<br>Proline
**VAR 111**	**3.354 - 3.373**	Proline
**VAR 112**	**3.346 - 3.352**	Proline<br>Tryptophan
**VAR 113**	**3.332 - 3.340**	1-methylhistidine

		Proline<br>Tryptophan
**VAR 114**	**3.313 - 3.325**	1-methylhistidine<br>Myoinositol<br>Phenylalanine<br>Tryptophan
**VAR 115**	**3.304 - 3.313**	1-methylhistidine<br>Phenylalanine<br>Tryptophan
**VAR 116**	**3.300 - 3.304**	Myoinositol
**VAR 117**	**3.291 - 3.300**	1-methylhistidine<br>Phenylalanine<br>Taurine<br>Lipids: $(O-CH_2-CH_2-N^+(C\mathbf{H_3})_3$ of PC and SM
**VAR 118**	**3.284 - 3.289**	Betaine<br>Myoinositol<br>Phenylalanine<br>Lipids: $(O-CH_2-CH_2-N^+(C\mathbf{H_3})_3$ of PC and SM
**VAR 119**	**3.278 - 3.284**	Glucose<br>Histidine<br>Taurine<br>Lipids: $(O-CH_2-CH_2-N^+(C\mathbf{H_3})_3$ of PC and SM
**VAR 120**	**3.270 - 3.278**	Arginine<br>Histidine<br>Taurine<br>Lipids: $(O-CH_2-CH_2-N^+(C\mathbf{H_3})_3$ of PC and SM
**VAR 121**	**3.263 - 3.270**	Arginine<br>Glucose<br>Lipids: $(O-CH_2-CH_2-N^+(C\mathbf{H_3})_3$ of PC and SM
**VAR 122**	**3.250 - 3.263**	Arginine<br>Glucose<br>Histidine<br>Lipids: $(O-CH_2-CH_2-N^+(C\mathbf{H_3})_3$ of PC and SM
**VAR 123**	**3.244 - 3.250**	1-methylhistidine<br>Carnitine<br>Histidine<br>Lipids: $(O-CH_2-CH_2-N^+(C\mathbf{H_3})_3$ of PC and SM
**VAR 124**	**3.236 - 3.244**	Lipids: $(O-CH_2-CH_2-N^+(C\mathbf{H_3})_3$ of PC and SM
**VAR 125**	**3.231 - 3.236**	1-methylhistidine<br>Tyrosine<br>Lipids: $(O-CH_2-CH_2-N^+(C\mathbf{H_3})_3$ of PC and SM
**VAR 126**	**3.227 - 3.231**	Cystine<br>Lipids: $(O-CH_2-CH_2-N^+(C\mathbf{H_3})_3$ of PC and SM
**VAR 127**	**3.224 - 3.227**	Tyrosine<br>Lipids: $(O-CH_2-CH_2-N^+(C\mathbf{H_3})_3$ of PC and SM
**VAR 128**	**3.217 - 3.222**	1-methylhistidine<br>Choline

		Tyrosine<br>Lipids: (O-CH_2-CH_2-N^+(C**H**$_3$)$_3$ of PC and SM
**VAR 129**	**3.211 - 3.217**	β-alanine<br>Tyrosine<br>Cystine<br>Lipids: (O-CH_2-CH_2-N^+(C**H**$_3$)$_3$ of PC and SM
**VAR 130**	**3.195 - 3.211**	1-methylhistidine<br>3-methylhistidine<br>β-alanine<br>Tyrosine<br>Cystine<br>Lipids: (O-CH_2-CH_2-N^+(C**H**$_3$)$_3$ of PC and SM
**VAR 131**	**3.187 - 3.193**	β-alanine<br>Cystine
**VAR 132**	**3.177 - 3.183**	3-methylhistidine<br>Histidine
**VAR 133**	**3.169 - 3.175**	3-methylhistidine
**VAR 134**	**3.135 - 3.169**	Histidine<br>Phenylalanine
**VAR 135**	**3.122 - 3.134**	Cysteine<br>Phenylalanine
**VAR 136**	**3.100 - 3.122**	3-methylhistidine<br>Cysteine
**VAR 137**	**3.090 - 3.100**	3-methylhistidine<br>Cysteine<br>Tyrosine
**VAR 138**	**3.077 - 3.090**	3-methyl-2-oxobutyrate<br>3-methylhistidine<br>Ornithine<br>Tyrosine
**VAR 139**	**3.066 - 3.077**	3-methyl-2-oxobutyrate<br>3-methylhistidine<br>Cysteine<br>Ornithine<br>Tyrosine
**VAR 140**	**3.060 - 3.066**	3-methyl-2-oxobutyrate<br>Creatinine<br>Cysteine<br>Ornithine
**VAR 141**	**3.053 - 3.060**	3-methyl-2-oxobutyrate<br>Creatine<br>Lysine<br>Tyrosine
**VAR 142**	**3.040 - 3.053**	3-methyl-2-oxobutyrate<br>4-aminobutyrate<br>Cysteine<br>Lysine

**VAR 143**	**3.035 - 3.040**	3-methyl-2-oxobutyrate<br>Cysteine<br>α-ketoglutarate
**VAR 144**	**3.029 - 3.035**	4-aminobutyrate<br>Lysine
**VAR 145**	**3.023 - 3.029**	3-methyl-2-oxobutyrate<br>α-ketoglutarate
**VAR 146**	**3.017 - 3.023**	4-aminobutyrate
**VAR 147**	**3.010 - 3.017**	α-ketoglutarate
**VAR 148**	**2.979 - 2.991**	Asparagine
**VAR 149**	**2.966 - 2.979**	3-methyl-2-oxopentanoate<br>N-acetylcysteine
**VAR 150**	**2.958 - 2.966**	3-methyl-2-oxopentanoate<br>Asparagine
**VAR 151**	**2.950 - 2.958**	Asparagine<br>N-acetylcysteine
**VAR 152**	**2.911 - 2.950**	3-methyl-2-oxopentanoate<br>N-acetylcysteine
**VAR 153**	**2.875 - 2.904**	Asparagine
**VAR 154**	**2.854 - 2.875**	Asparagine<br>Lipids: =CH-C$\mathbf{H_2}$-CH= in FAC
**VAR 155**	**2.812 - 2.854**	Aspartate<br>Lipids: =CH-C$\mathbf{H_2}$-CH= in FAC
**VAR 156**	**2.762 - 2.812**	Lipids: =CH-C$\mathbf{H_2}$-CH= in FAC
**VAR 157**	**2.757 - 2.762**	Sarcosine<br>Lipids: =CH-C$\mathbf{H_2}$-CH= in FAC
**VAR 158**	**2.741 - 2.757**	Lipids: =CH-C$\mathbf{H_2}$-CH= in FAC
**VAR 159**	**2.724 - 2.741**	NI 18<br>Lipids: =CH-C$\mathbf{H_2}$-CH= in FAC
**VAR 160**	**2.718 - 2.724**	Aspartate<br>Lipids: =CH-C$\mathbf{H_2}$-CH= in FAC
**VAR 161**	**2.712 - 2.718**	Lipids: =CH-C$\mathbf{H_2}$-CH= in FAC
**VAR 162**	**2.702 - 2.712**	Aspartate<br>Citrate
**VAR 163**	**2.690 - 2.694**	Aspartate
**VAR 164**	**2.673 - 2.685**	Aspartate<br>Citrate<br>Methionine
**VAR 165**	**2.647 - 2.668**	Methionine
**VAR 166**	**2.615 - 2.634**	4-methyl-2-oxovalerate
**VAR 167**	**2.571 - 2.592**	β-alanine
**VAR 168**	**2.533 - 2.571**	β-alanine<br>Citrate<br>Pyroglutamate
**VAR 169**	**2.501 - 2.533**	Glutamine<br>Pyroglutamate

**VAR 170**	**2.484 - 2.501**	Carnitine<br>Glutamine<br>Pyroglutamate
**VAR 171**	**2.476 - 2.484**	Glutamine
**VAR 172**	**2.450 - 2.476**	Carnitine<br>Glutamine<br>Hydroxyproline<br>α-ketoglutarate
**VAR 173**	**2.439 - 2.450**	Carnitine<br>Glutamine<br>Hydroxyproline<br>α-ketoglutarate<br>β-Hydroxybutyrate
**VAR 174**	**2.427 - 2.439**	Carnitine<br>Glutamine<br>Hydroxyproline<br>Pyroglutamate<br>β-Hydroxybutyrate
**VAR 175**	**2.418 - 2.427**	Carnitine<br>Pyroglutamate<br>Succinate<br>β-Hydroxybutyrate
**VAR 176**	**2.405 - 2.418**	Glutamate<br>Pyroglutamate<br>β-Hydroxybutyrate
**VAR 177**	**2.380 - 2.405**	3-hydroxy-3-methylbutyrate<br>Glutamate<br>Oxaloacetate<br>Proline<br>Pyruvate
**VAR 178**	**2.349 - 2.380**	Glutamate<br>Proline
**VAR 179**	**2.337 - 2.349**	Glutamate<br>Proline<br>β-Hydroxybutyrate
**VAR 180**	**2.324 - 2.337**	4-aminobutyrate<br>Glutamate<br>Valine<br>β-Hydroxybutyrate
**VAR 181**	**2.312 - 2.324**	4-aminobutyrate<br>Valine<br>β-Hydroxybutyrate
**VAR 182**	**2.305 - 2.312**	Valine<br>β-Hydroxybutyrate
**VAR 183**	**2.294 - 2.305**	4-aminobutyrate<br>Valine<br>Acetoacetate

**VAR 184**	**2.282 - 2.294**	Valine
**VAR 185**	**2.264 - 2.282**	Valine<br>Lipids: -C**H**$_2$-C=O or -C**H**$_2$-CH=CH- in FAC
**VAR 186**	**2.254 - 2.264**	Lipids: -C**H**$_2$-C=O or -C**H**$_2$-CH=CH- in FAC
**VAR 187**	**2.221 - 2.254**	Methionine<br>Lipids: -C**H**$_2$-C=O or -C**H**$_2$-CH=CH- in FAC
**VAR 188**	**2.208 - 2.221**	Methionine
**VAR 189**	**2.151 - 2.208**	Glutamate<br>Glutamine<br>Hydroxyproline<br>Methionine
**VAR 190**	**2.128 - 2.151**	4-methyl-2-oxovalerate<br>Glutamate<br>Glutamine<br>Methionine
**VAR 191**	**2.114 - 2.128**	4-methyl-2-oxovalerate<br>Glutamate<br>Glutamine<br>Methionine<br>Proline
**VAR 192**	**2.102 - 2.114**	4-methyl-2-oxovalerate<br>Glutamate<br>Glutamine<br>Methionine<br>Proline<br>Lipids: -C**H**$_2$-CH=CH- in FAC; C**H**$_3$ of NAG
**VAR 193**	**2.078 - 2.102**	Glutamate<br>N-acetylcysteine<br>Proline<br>Pyroglutamate<br>Lipids: -C**H**$_2$-CH=CH- in FAC; C**H**$_3$ of NAG
**VAR 194**	**2.042 - 2.078**	2-hydroxy-3-methylbutyrate<br>Glutamate<br>Proline<br>Pyroglutamate<br>Lipids: -C**H**$_2$-CH=CH- in FAC; C**H**$_3$ of NAG
**VAR 195**	**2.013 - 2.042**	2-hydroxy-3-methylbutyrate<br>Isoleucine<br>Proline<br>Pyroglutamate<br>Lipids: -C**H**$_2$-CH=CH- in FAC; C**H**$_3$ of NAG
**VAR 196**	**2.001 - 2.013**	Isoleucine<br>Ornithine<br>Proline<br>Lipids: -C**H**$_2$-CH=CH- in FAC; C**H**$_3$ of NAG

**VAR 197**	**1.967 - 2.001**	Arginine<br>Isoleucine<br>Ornithine<br>Proline<br>2-aminobutyrate<br>Lipids: -C$\mathbf{H_2}$-CH=CH- in FAC; C$\mathbf{H_3}$ of NAG
**VAR 198**	**1.949 - 1.967**	Arginine<br>Lysine<br>Ornithine<br>2-aminobutyrate
**VAR 199**	**1.906 - 1.949**	4-aminobutyrate<br>Acetate<br>Arginine<br>Lysine<br>Ornithine<br>2-aminobutyrate
**VAR 200**	**1.823 - 1.906**	4-aminobutyrate<br>Arginine<br>Lysine<br>Ornithine<br>2-aminobutyrate
**VAR 201**	**1.800 - 1.823**	Ornithine
**VAR 202**	**1.729 - 1.800**	2-hydroxybutyrate<br>3-methyl-2-oxopentanoate<br>Arginine<br>Leucine<br>Lysine<br>Ornithine
**VAR 203**	**1.717 - 1.729**	2-hydroxybutyrate<br>3-methyl-2-oxopentanoate<br>Arginine<br>Leucine<br>Lysine
**VAR 204**	**1.678 - 1.717**	2-hydroxybutyrate<br>3-methyl-2-oxopentanoate<br>Arginine<br>Leucine
**VAR 205**	**1.655 - 1.678**	2-hydroxybutyrate<br>Arginine
**VAR 206**	**1.650 - 1.655**	Arginine
**VAR 207**	**1.630 - 1.650**	2-hydroxybutyrate<br>Arginine<br>Lipids: -C$\mathbf{H_2}$-CH_2-C=O or -C$\mathbf{H_2}$-CH_2-CH=CH- in FAC
**VAR 208**	**1.572 - 1.630**	Lipids: -C$\mathbf{H_2}$-CH_2-C=O or -C$\mathbf{H_2}$-CH_2-CH=CH- in FAC
**VAR 209**	**1.535 - 1.572**	Lysine<br>Lipids: -C$\mathbf{H_2}$-CH_2-C=O or -C$\mathbf{H_2}$-CH_2-CH=CH- in FAC

**VAR 210**	**1.509 - 1.535**	Isoleucine<br>Lysine
**VAR 211**	**1.489 - 1.509**	Alanine<br>Isoleucine<br>Lysine
**VAR 212**	**1.446 - 1.489**	3-methyl-2-oxopentanoate<br>Isoleucine<br>Lysine
**VAR 213**	**1.408 - 1.446**	3-methyl-2-oxopentanoate<br>Lysine<br>NI 19
**VAR 214**	**1.358 - 1.405**	Lipids: CH_3-(C**H_2**)$_n$- in FAC
**VAR 215**	**1.338 - 1.358**	Lactate<br>Threonine<br>Lipids: CH_3-(C**H_2**)$_n$- in FAC
**VAR 216**	**1.296 - 1.338**	Lipids: CH_3-(C**H_2**)$_n$- in FAC
**VAR 217**	**1.289 - 1.296**	Isoleucine<br>Lipids: CH_3-(C**H_2**)$_n$- in FAC
**VAR 218**	**1.285 - 1.289**	3-hydroxy-3-methylbutyrate<br>Isoleucine<br>Lipids: CH_3-(C**H_2**)$_n$- in FAC
**VAR 219**	**1.226 - 1.285**	Isoleucine<br>Lipids: CH_3-(C**H_2**)$_n$- in FAC
**VAR 220**	**1.211 - 1.226**	Isoleucine<br>β-Hydroxybutyrate<br>Lipids: CH_3-(C**H_2**)$_n$- in FAC
**VAR 221**	**1.200 - 1.211**	Lipids: CH_3-(C**H_2**)$_n$- in FAC
**VAR 222**	**1.182 - 1.198**	Isopropanol
**VAR 223**	**1.128 - 1.151**	3-methyl-2-oxobutyrate
**VAR 224**	**1.103 - 1.123**	3-methyl-2-oxopentanoate
**VAR 225**	**1.078 - 1.101**	NI 20
**VAR 226**	**1.052 - 1.072**	Valine
**VAR 227**	**1.020 - 1.039**	Isoleucine
**VAR 228**	**1.008 - 1.020**	Valine<br>2-aminobutyrate
**VAR 229**	**1.001 - 1.008**	Valine
**VAR 230**	**0.996 - 1.001**	2-aminobutyrate
**VAR 231**	**0.984 - 0.996**	2-hydroxy-3-methylbutyrate<br>Leucine<br>2-aminobutyrate
**VAR 232**	**0.976 - 0.984**	2-hydroxy-3-methylbutyrate<br>Leucine
**VAR 233**	**0.964 - 0.976**	Isoleucine<br>Leucine
**VAR 234**	**0.938 - 0.964**	4-methyl-2-oxovalerate<br>Isoleucine

**VAR 235**	**0.929 - 0.938**	2-hydroxybutyrate
**VAR 236**	**0.882 - 0.929**	2-hydroxybutyrate<br>3-methyl-2-oxopentanoate<br>Lipids: C**H_3**-$(CH_2)_n$- in FAC
**VAR 237**	**0.800 - 0.882**	2-hydroxy-3-methylbutyrate<br>Lipids: C**H_3**-$(CH_2)_n$- in FAC

Table S2: Overview of the intrasample variability of the 237 integration regions. Shown is the small relative standard deviation (%RSD) of each variable based on the analysis of 12 identical plasma samples from a plasma pool. The signal of maleic acid was used to normalize all integration regions. Integration regions having an %RSD >10% are colored in red and were excluded from further statistical analysis. RSD: Relative standard deviation; VAR: variable.

Assigned integration number (variable)	RSD(%)	Assigned integration number (variable)	RSD(%)
VAR 001	11.75	VAR 042	1.62
VAR 002	6.10	VAR 043	2.76
VAR 003	6.79	VAR 044	4.38
VAR 004	5.72	VAR 045	1.60
VAR 005	5.76	VAR 046	2.27
VAR 006	6.21	VAR 047	3.07
VAR 007	5.57	VAR 048	1.69
VAR 008	5.07	VAR 049	3.61
VAR 009	13.59	VAR 050	2.52
VAR 010	7.13	VAR 051	1.59
VAR 011	2.70	VAR 052	2.41
VAR 012	2.93	VAR 053	4.07
VAR 013	4.75	VAR 054	2.43
VAR 014	6.38	VAR 055	2.27
VAR 015	2.82	VAR 056	4.15
VAR 016	3.73	VAR 057	1.24
VAR 017	4.20	VAR 058	3.36
VAR 018	7.42	VAR 059	2.25
VAR 019	1.95	VAR 060	2.96
VAR 020	5.22	VAR 061	2.74
VAR 021	336.77	VAR 062	0.99
VAR 022	18.02	VAR 063	0.94
VAR 023	5.03	VAR 064	0.87
VAR 024	13.9	VAR 065	2.31
VAR 025	1.41	VAR 066	0.97
VAR 026	6.94	VAR 067	3.87
VAR 027	55.22	VAR 068	2.24
VAR 028	4.56	VAR 069	2.43
VAR 029	18.82	VAR 070	1.00
VAR 030	9.83	VAR 071	1.52
VAR 031	13.26	VAR 072	1.55
VAR 032	8.08	VAR 073	1.15
VAR 033	5.38	VAR 074	2.48
VAR 034	3.25	VAR 075	1.10
VAR 035	3.07	VAR 076	1.04
VAR 036	3.71	VAR 077	6.73
VAR 037	3.45	VAR 078	1.84
VAR 038	2.68	VAR 079	1.78
VAR 039	1.49	VAR 080	2.01

**VAR 040**	1.93	**VAR 081**	1.63
**VAR 041**	1.27	**VAR 082**	3.00
**VAR 083**	2.78	**VAR 129**	3.13
**VAR 084**	3.19	**VAR 130**	5.33
**VAR 085**	3.14	**VAR 131**	7.77
**VAR 086**	2.39	**VAR 132**	6.94
**VAR 087**	2.45	**VAR 133**	4.42
**VAR 088**	1.54	**VAR 134**	7.16
**VAR 089**	3.39	**VAR 135**	6.93
**VAR 090**	2.87	**VAR 136**	6.56
**VAR 091**	1.93	**VAR 137**	4.51
**VAR 092**	2.23	**VAR 138**	5.14
**VAR 093**	0.90	**VAR 139**	3.39
**VAR 094**	1.32	**VAR 140**	2.12
**VAR 095**	6.14	**VAR 141**	2.63
**VAR 096**	1.54	**VAR 142**	2.77
**VAR 097**	1.46	**VAR 143**	3.05
**VAR 098**	4.70	**VAR 144**	3.26
**VAR 099**	1.71	**VAR 145**	4.51
**VAR 100**	1.27	**VAR 146**	4.10
**VAR 101**	3.80	**VAR 147**	3.88
**VAR 102**	2.15	**VAR 148**	4.66
**VAR 103**	1.74	**VAR 149**	2.33
**VAR 104**	3.24	**VAR 150**	5.06
**VAR 105**	5.54	**VAR 151**	3.61
**VAR 106**	1.21	**VAR 152**	4.00
**VAR 107**	1.83	**VAR 153**	6.54
**VAR 108**	5.20	**VAR 154**	4.29
**VAR 109**	3.57	**VAR 155**	1.87
**VAR 110**	3.85	**VAR 156**	2.60
**VAR 111**	3.85	**VAR 157**	3.75
**VAR 112**	4.67	**VAR 158**	1.85
**VAR 113**	7.07	**VAR 159**	2.74
**VAR 114**	4.07	**VAR 160**	4.81
**VAR 115**	6.26	**VAR 161**	2.19
**VAR 116**	7.01	**VAR 162**	2.41
**VAR 117**	3.12	**VAR 163**	8.03
**VAR 118**	4.67	**VAR 164**	2.05
**VAR 119**	1.15	**VAR 165**	5.74
**VAR 120**	2.74	**VAR 166**	9.20
**VAR 121**	1.46	**VAR 167**	7.22
**VAR 122**	2.95	**VAR 168**	3.70
**VAR 123**	2.47	**VAR 169**	6.31
**VAR 124**	2.05	**VAR 170**	3.42
**VAR 125**	2.63	**VAR 171**	1.39
**VAR 126**	1.82	**VAR 172**	2.05

**VAR 127**	7.30	**VAR 173**	3.41
**VAR 128**	2.76	**VAR 174**	4.11
**VAR 175**	3.33	**VAR 207**	3.57
**VAR 176**	3.41	**VAR 208**	3.88
**VAR 177**	3.94	**VAR 209**	2.37
**VAR 178**	2.56	**VAR 210**	3.28
**VAR 179**	3.27	**VAR 211**	1.33
**VAR 180**	2.27	**VAR 212**	2.72
**VAR 181**	2.84	**VAR 213**	2.25
**VAR 182**	3.97	**VAR 214**	2.33
**VAR 183**	2.02	**VAR 215**	3.11
**VAR 184**	4.94	**VAR 216**	1.72
**VAR 185**	4.80	**VAR 217**	3.85
**VAR 186**	3.61	**VAR 218**	3.51
**VAR 187**	2.31	**VAR 219**	3.97
**VAR 188**	1.88	**VAR 220**	1.22
**VAR 189**	1.83	**VAR 221**	2.49
**VAR 190**	3.22	**VAR 222**	2.76
**VAR 191**	2.28	**VAR 223**	2.93
**VAR 192**	2.57	**VAR 224**	4.45
**VAR 193**	1.97	**VAR 225**	4.24
**VAR 194**	2.72	**VAR 226**	1.52
**VAR 195**	1.72	**VAR 227**	2.39
**VAR 196**	1.87	**VAR 228**	1.61
**VAR 197**	1.94	**VAR 229**	1.18
**VAR 198**	1.80	**VAR 230**	2.06
**VAR 199**	4.64	**VAR 231**	1.50
**VAR 200**	5.93	**VAR 232**	2.68
**VAR 201**	2.50	**VAR 233**	2.05
**VAR 202**	3.37	**VAR 234**	2.25
**VAR 203**	2.67	**VAR 235**	3.66
**VAR 204**	3.34	**VAR 236**	1.87
**VAR 205**	3.45	**VAR 237**	2.50
**VAR 206**	4.63		

Table S3: Overview of the 30 variables with the highest total VIP-value based on the OPLS-DA model constructed with 78 variables. The variables are shown with decreasing VIP-value. Red and green arrows indicate respectively a decrease or increase in integration values of these variables in lung cancer patients compared to healthy controls. VAR: variable (assigned integration number); VIP: variable importance in projection.

Assigned integration number (variable)	Total VIP-value	Assigned integration number (variable)	Total VIP-value
**VAR 195**	3.61 ↓	**VAR 061**	1.19 ↑
**VAR 218**	2.28 ↓	**VAR 034**	1.18 ↓
**VAR 186**	2.19 ↓	**VAR 080**	1.10 ↑
**VAR 185**	2.13 ↓	**VAR 126**	1.10 ↓
**VAR 187**	2.03 ↓	**VAR 062**	1.10 ↑
**VAR 156**	1.88 ↓	**VAR 082**	1.03 ↑
**VAR 057**	1.83 ↑	**VAR 067**	1.03 ↑
**VAR 222**	1.83 ↑	**VAR 127**	0.95 ↓
**VAR 042**	1.57 ↓	**VAR 081**	0.95 ↑
**VAR 059**	1.54 ↑	**VAR 055**	0.87 ↑
**VAR 066**	1.31 ↑	**VAR 207**	0.86 ↓
**VAR 035**	1.27 ↓	**VAR 202**	0.85 ↑
**VAR 060**	1.25 ↑	**VAR 090**	0.85 ↑
**VAR 227**	1.25 ↑	**VAR 083**	0.84 ↑
**VAR 209**	1.22 ↓	**VAR 078**	0.82 ↑

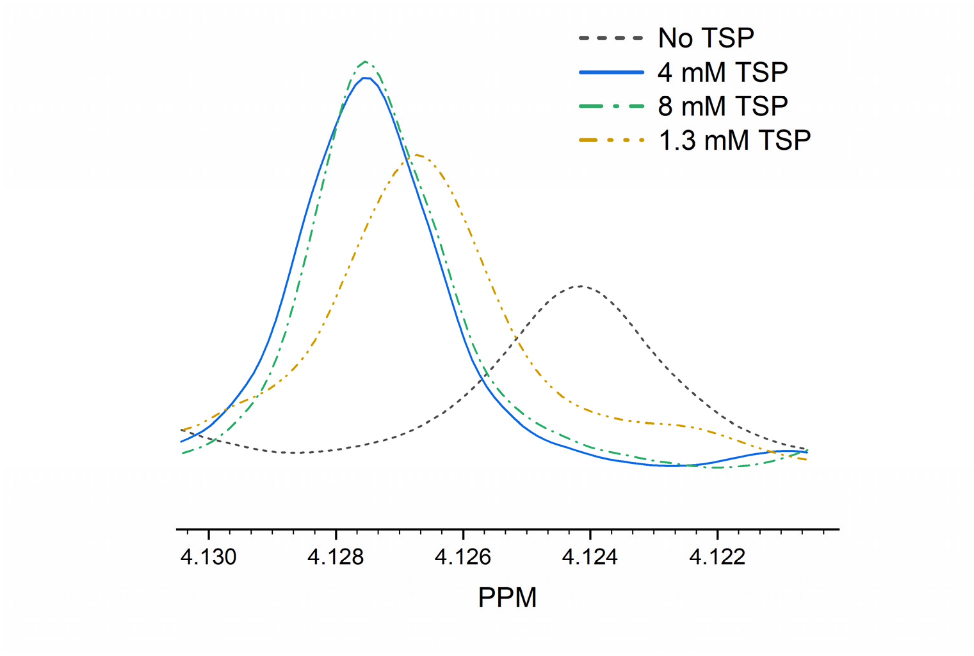

Figure S1: Zoom-in of a lactate signal (of the $^{\alpha}$CH quadruplet) upon the addition of different amounts of TSP. After addition of 4 mM TSP, stable chemical shifts and signal intensities are reached. Higher TSP concentrations do not influence the spectrum further. TSP: trimethylsilyl-2,2,3,3-tetradeuteropropionic acid.

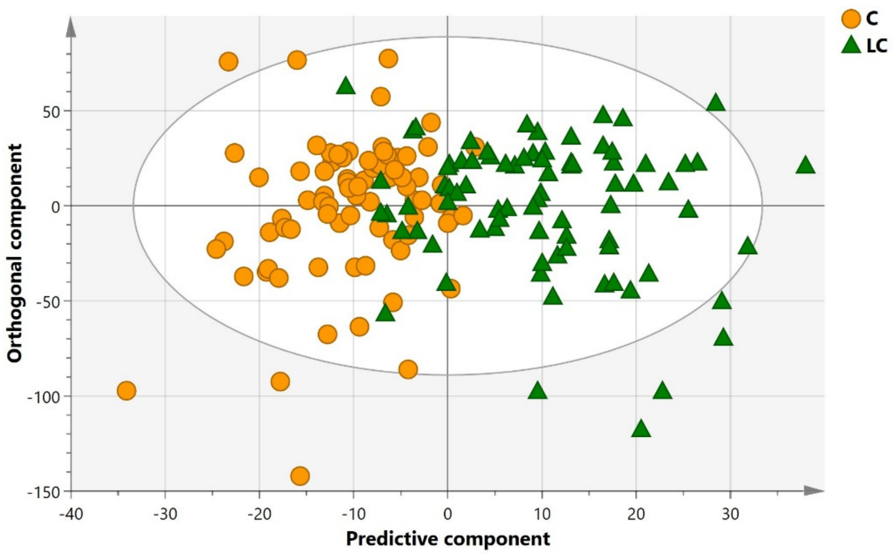

Figure S2: Orthogonal partial least squares discriminant analysis (OPLS-DA) classifier of the training cohort consisting of 80 controls and 80 lung cancer patients, using 221 variables. Model parameter values of R^2X(cum), R^2Y(cum) and Q^2(cum) are 0.833, 0.577 and 0.337 respectively. From this model, values of the loadings and jack knife interval are extracted for each variable in order to perform data reduction. C: controls; LC: lung cancer patients.

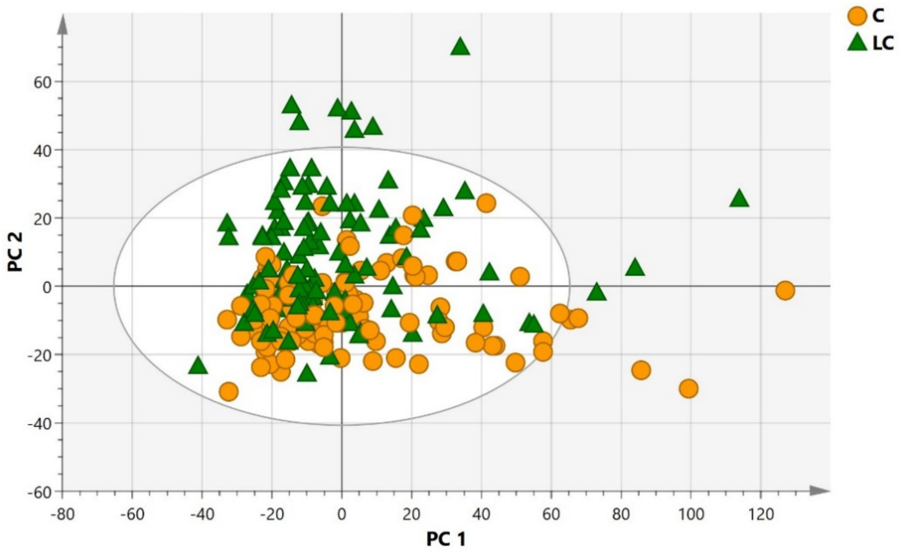

Figure S3: Principal component analysis (PCA) of all 232 subjects (using the reduced dataset of 78 variables) from the training and validation cohort showing a clustering trend for the controls and lung cancer patients. Using only the first two principal components, the PCA model shows a R^2 value of 0.774 and a Q^2 value of 0.749. C: controls; LC: lung cancer patients; PC: principal component.

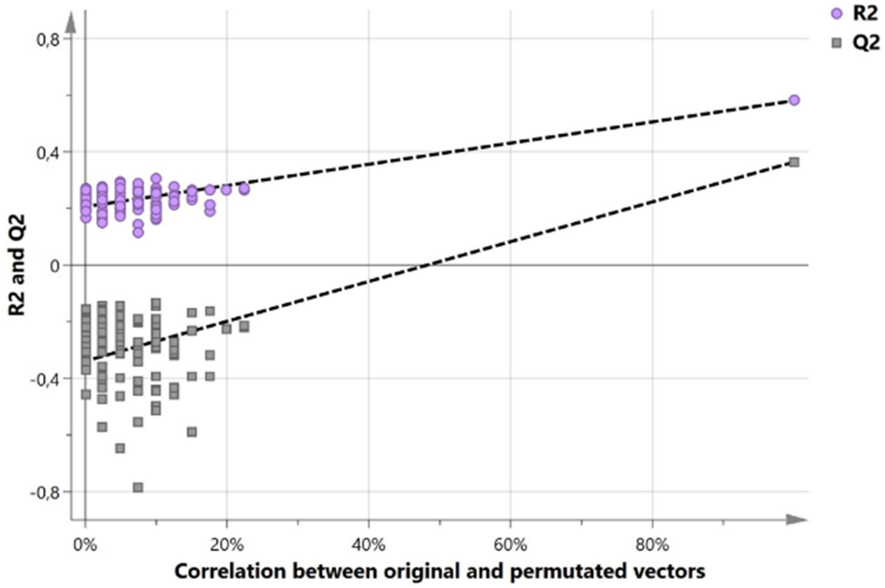

Figure S4: Permutation test of the training model using 100 permutations.
